# Developing an intervention to optimise the outcome of cardiac surgery in people with diabetes: the OCTOPuS pilot study

**DOI:** 10.1186/s40814-021-00887-z

**Published:** 2021-08-17

**Authors:** Richard I. G. Holt, Katharine Barnard-Kelly, Giorgos Dritsakis, Kerensa I. Thorne, Lauren Cohen, Elizabeth Dixon, Mayank Patel, Philip Newland-Jones, Helen Partridge, Suvitesh Luthra, Sunil Ohri, Kareem Salhiyyah, Jo Picot, John Niven, Andrew Cook, on behalf of the OCTOPuS study group, Theodore Velissaris, Paula Johnson, Rita Trodden, Mark Green, Jana Rojkova, Thea Sass, Jo Stanley, Alexandra Collier, Susi Renz, Jess Boxall, Josh Northey, Louise Stanton, Amy Whitehead, Ketan Dhatariya, Debbie Stanisstreet, Kamran Baig, Merryn Voysey, Donna Drinkwater, Joanne Lord, Jonathan Shepherd

**Affiliations:** 1 Human Development and Health, Faculty of Medicine, University of Southampton, Southampton, UK; 2 Southampton National Institute for Health Research Biomedical Research Centre, University Hospital Southampton NHS Foundation Trust, Southampton, UK; 3 Barnard Health–Health Psychology Research, Fareham, UK; 4 Faculty of Health & Social Science, Bournemouth University, Poole, UK; 5 Clinical Trials Unit, Faculty of Medicine, University of Southampton, Southampton, UK; 6 Diabetes Department, University Hospital Southampton, Southampton, UK; 7 Royal Bournemouth and Christchurch Hospitals NHS Foundation Trust, Bournemouth, UK; 8 Division of Cardiac Surgery, Wessex Cardiothoracic Centre, University Hospital Southampton, Southampton, UK; 9 Middle East University, Amman, Jordan; 10 Southampton Health Technology Assessments Centre, University of Southampton, Southampton, UK

**Keywords:** Diabetes, HbA_1c_, Cardiothoracic surgery, In-patient, Surgical outcomes, Service provision

## Abstract

**Background:**

Cardiothoracic surgical outcomes are poorer in people with diabetes compared with those without diabetes. There are two important uncertainties in the management of people with diabetes undergoing major surgery: (1) how to improve diabetes management in the weeks leading up to an elective procedure and (2) whether that improved management leads to improved postoperative outcomes. The aim of this study was to develop and pilot a specialist diabetes team-led intervention to improve surgical outcomes in people with diabetes.

**Design:**

Open pilot feasibility study

**Setting:**

Diabetes and cardiothoracic surgery departments, University Hospital Southampton NHS Foundation Trust

**Participants:**

Seventeen people with diabetes undergoing cardiothoracic surgery

**Intervention:**

Following two rapid literature reviews, a prototype intervention was developed based on a previously used nurse-led outpatient intervention and tested.

**Primary outcome:**

Feasibility and acceptability of delivering the intervention

**Secondary outcomes:**

Biomedical data were collected at baseline and prior to surgery. We assessed how the intervention was used. In depth qualitative interviews with participants and healthcare professionals were used to explore perceptions and experiences of the intervention and how it might be improved.

**Results:**

Thirteen of the 17 people recruited completed the study and underwent cardiothoracic surgery. All components of the OCTOPuS intervention were used, but not all parts were used for all participants. Minor changes were made to the intervention as a result of feedback from the participants and healthcare professionals. Median (IQR) HbA_1c_ was 10 mmol/mol (3, 13) lower prior to surgery than at baseline.

**Conclusion:**

This study has shown that it is possible to develop a clinical pathway to improve diabetes management prior to admission. The clinical and cost-effectiveness of this intervention will now be tested in a multicentre randomised controlled trial in cardiothoracic centres across the UK.

**Trial registration:**

ISRCTN; ISRCTN10170306. Registered 10 May 2018.

**Supplementary Information:**

The online version contains supplementary material available at 10.1186/s40814-021-00887-z.

## Key messages regarding feasibility

- What uncertainties existed regarding the feasibility?

There are two important uncertainties in the management of people with suboptimally managed diabetes undergoing major surgery: (1) how to improve diabetes management in the weeks leading up to an elective procedure and (2) whether that improved management is reflected in improved outcomes post-surgery.

- What are the key feasibility findings?

The study demonstrates that a preoperative intervention designed to improve the surgical outcomes of people with suboptimally managed diabetes undergoing cardiothoracic surgery was feasible and acceptable to patients and healthcare professionals.

- What are the implications of the feasibility findings for the design of the main study?

The intervention requires close collaboration between cardiothoracic and diabetes healthcare professionals to ensure a smooth care pathway. Further changes may be required because of the new ways of delivering care because of COVID-19.

## Introduction

The prevalence of cardiovascular disease is increased approximately 2-fold in people with diabetes after adjustment for other cardiovascular risk factors [1]. It affects approximately a third of all people with type 2 diabetes and is responsible for over 50% of deaths [2]. As coronary heart disease tends to be more diffuse affecting multiple vessels in people with diabetes, coronary artery bypass grafting is often the preferred method for re-vascularisation. Approximately 30–40% of people undergoing open cardiac surgery have diabetes [3].

Surgical outcomes are worse in people with diabetes with an up to three-fold higher risk of postoperative complications including poor healing, wound complications, and renal dysfunction [4, 5]. These complications are associated with longer hospital stay and higher readmission rates. The reasons underlying the poorer outcomes are multiple but include hyperglycaemia, dyslipidaemia and obesity. Although national and international groups have published detailed diabetes management guidelines to improve surgical outcomes in people with diabetes [6–8], many people with diabetes are poorly prepared for surgery. In the E-CABG registry, 54% of people with type 2 diabetes treated with non-insulin medications and 67% of those with insulin-treated diabetes had an HbA_1c_ above 53 mmol/mol (7.0%) prior to cardiac surgery [5].

Although the means of improving glycaemic management are well-known, currently, there are no systematic strategies to improve diabetes management prior to surgery, and this is dependent on individual surgeon preference and liaison with the patient’s general practitioner or diabetes team. Although the general practitioner can refer people to a diabetes specialist team for a review of the patient’s diabetes, this process is often too slow to allow time to make a meaningful difference to the HbA_1c_ before surgery.

A recent survey of cardiothoracic surgical practice across the UK found that only 44% of surgeons routinely measured HbA_1c_ prior to surgery, and only 19% have a threshold above which they would not operate [9]. The survey indicated that there was only limited perioperative management of diabetes, and practice depended on individual surgeons rather than local policy.

There are two important uncertainties in the management of people with suboptimally managed diabetes undergoing major surgery: (1) how to improve diabetes management in the weeks leading up to an elective procedure and (2) whether that improved management is reflected in improved outcomes post-surgery. The overarching aim of the Optimising Cardiac Surgery Outcomes in People with Diabetes (OCTOPuS) project is to develop and test whether a preoperative out-patient intervention to improve diabetes management improves cardiac surgical outcomes. This paper reports the development and pilot testing of the intervention (Additional file 1 contains the pilot trial protocol).

## Methods

### Development of the intervention

The intervention was developed according to the MRC Framework for Complex Interventions [10].

### Rapid literature review

The project team undertook two rapid literature reviews to identify (1) the modifiable factors associated with poor surgical outcomes in people with diabetes and (2) to determine what pre-admission interventions to improve surgical outcomes had been evaluated previously. Searches were undertaken using the MEDLINE, Embase and The Cochrane Library (including CDSR and DARE) databases between January 2000 and January 2018 (Additional file 2).

For the first review, we reviewed eight previously published systematic reviews or meta-analyses [11–18]. Seven of these reported on glycaemic management [11–17]. Two of the three papers reporting on HbA_1c_ confirmed that elevated HbA_1c_ was associated with poorer surgical outcomes [11–13]. However, these reviews had a critically low overall confidence rating when assessed by AMSTAR2 criteria. The eighth systematic review, also with a critically low overall confidence rating, reported that the preoperative use of renin-angiotensin system blockers was associated with lower early mortality [18].

The second review identified four relevant studies published in five papers [19–23]. Three studies reported that a preoperative intervention led to reduced preoperative HbA_1c_ and/or glucose [20–23], and although one reported that the intervention shortened the length of hospital stay [22], another found no significant difference [19]. Although readmission was reduced in one study, this could not be attributed to the intervention [22]. Previous studies have identified smoking and obesity as risk factors for poor surgical outcomes in the general population [24–26].

### Development of a prototype intervention

Our intervention, known as OCTOPuS, was based on an established nurse-led outpatient intervention used by the Royal Bournemouth Hospital diabetes team. This is delivered around 3 months before surgery to people with suboptimally managed diabetes and achieved a reduction in mean HbA_1c_ from 85 at first referral to 74 mmol/mol on admission for surgery. This was associated with a reduced mean length of stay from 5.9 to 3 days. During the same period, the length of stay for those without diabetes remained constant at 5 days [27].

The research team, comprising diabetes healthcare professionals (including staff from Bournemouth), cardiac surgeons, an anaesthetist, trial investigators and people with diabetes who had undergone surgery, discussed the Bournemouth intervention. Further input was obtained from the trial oversight committee, public and patient involvement advisory group and local branch of Diabetes UK. The advice for glycaemic management was adapted from the Bournemouth intervention, and we added further content to address hypertension, dyslipidaemia, smoking, weight management, exercise and social support from friends, relatives and carers (Additional file 3). All clinical advice represented current best practice based on NICE guidance or the EASD-ADA position statement on the management of type 2 diabetes [28, 29]. Having agreed the content, we manualised the intervention so it could be used by other UK surgical centres (Additional file 3).

### Pilot of the OCTOPuS intervention

After creating the manual, the intervention was piloted to test its acceptability and feasibility for people with diabetes awaiting cardiac surgery and the healthcare professionals delivering the intervention to allow further adaptations as necessary.

### Study design

The study was a single centre, feasibility study to pilot an outpatient manualised intervention to assess its acceptability to people with suboptimally managed diabetes undergoing elective cardiothoracic surgery and health professionals. Participation in the trial ended 30 days post-surgery.

### Setting

The study was conducted at the University Hospital Southampton NHS Foundation Trust.

### Participants

Initially, adults (aged 18–75 years) with suboptimally managed type 1 diabetes or type 2 diabetes were invited to participate. Suboptimally managed diabetes was defined as an HbA_1c_ >53 mmol/mol (7%). There was no upper limit of HbA_1c_. A substantial amendment was approved on 11 October 2019 to include people over the age of 75 years, when it became apparent that a significant number of people with diabetes in this age group could potentially benefit from the intervention. A higher HbA_1c_ threshold of ≥64 mmol/mol was used for older people because of the higher risk of hypoglycaemia and its consequences in this age group.

A traditional approach to seeking consent to participate in the research at the outpatient appointment and giving patients at least 24 h to consider and discuss the trial before making a decision presented a challenge because it would require an extra visit to a potentially distant hospital. Instead, cardiothoracic outpatient appointment lists were scrutinised by research nurses ahead of appointments, to identify people with diabetes referred for consideration of cardiac surgery. An information sheet explaining the study was sent to those who appeared eligible. Before the outpatient appointment, a researcher telephoned the prospective participant to discuss the study, allowing sufficient time for reflection and discussion (at least 24 h) before the outpatient appointment. The information sheet included contact details to opt out if the patient did not want to be contacted about the trial. Foss et al. previously used this method and demonstrated that patients receiving telephone-based counselling about a trial showed similar levels of comprehension to those being counselled face-to-face [30].

During the cardiothoracic outpatient appointment, if a decision to proceed to elective cardiac surgery was made, and the patient was willing to participate, a research nurse conducted a more detailed interview during which the study was discussed according to the needs of the patient, and final exclusion criteria checked and written consent given. A capillary glucose sample was obtained to measure the HbA_1c_ using a near patient test at the cardiothoracic outpatient appointment to check eligibility. Participants were not paid for participation in this study. Screening failures were recorded.

Exclusion criteria were as follows: active malignancy or other illnesses or conditions that would preclude engagement with OCTOPuS, pregnancy, previous cardiac surgery, the need for urgent surgery (<2 months from the out-patient visit), known haemoglobinopathies.

### Study procedures

#### Intervention

Full details of the OCTOPuS intervention are given in Additional file 3. The intervention was designed to be delivered by healthcare professionals with expertise in diabetes. These individuals could be diabetes nurse specialists, consultant physicians, pharmacists or dietitians. As the initial consultation was likely to result in changes to medication, the OCTOPuS practitioners needed to be prescribers or have easy access to a prescriber. All OCTOPuS practitioners received additional specific training about the OCTOPuS intervention.

The intervention ran from the point at which patients were accepted for cardiac surgery until surgery took place (anticipated to be 2–3 months later based on current waiting lists). After an initial consultation, participants received regular telephone review with the OCTOPuS practitioner, at least once a fortnight until surgery, providing an opportunity to offer encouragement and support, and address any matters arising.

The OCTOPuS intervention comprised seven components: glucose management, lipid management, hypertension management, weight management, exercise, smoking cessation and support from spouses, other relatives or friends. Given the short duration of the intervention and limited opportunity for contacts, we were aware that it would not be feasible to implement all actions and changes suggested by the manual. However, we envisaged that the OCTOPuS practitioner would work with the patient to implement as much as possible to bring the patient to the best clinical status prior to surgery. The intervention involved an initial assessment and management plan, which would ideally take place as soon as possible after listing for surgery, followed by regular fortnightly telephone consultations. When making treatment decisions, OCTOPuS practitioners were expected to consider the individual needs, preferences and values of the patients, and the OCTOPuS manual did not override the responsibility to make clinical decisions appropriate to individual circumstances.

Where necessary, the OCTOPuS practitioner liaised with local services, e.g. the participant’s GP or a dietitian, to facilitate delivery.

##### Glucose management

A key goal of the intervention was to lower HbA_1c_ (Section 2.2 Additional file 3). During the initial consultation, the participant’s diabetes management was discussed, as well as the likely benefits that improved glycaemic levels would provide prior to surgery. The importance of lifestyle modification was discussed and reinforced. Where the participant was not already monitoring blood glucose, this was initiated. Antidiabetes medications were adjusted where necessary, with particular emphasis on using SGLT2 inhibitors or GLP-1 receptor agonists that have demonstrable cardiovascular benefits. Where necessary, insulin was initiated or doses or regimen adjusted. The practitioner and participant agreed on an action plan, tailored to the individual’s needs and ability according to the manual.

##### Lipid management

Although it was envisaged that most participants would already be taking lipid-lowering therapy, if they were not taking a statin, the OCTOPuS practitioner discussed the benefits of statin therapy and recommended that this was initiated if there were no contraindications or the person had previously not tolerated treatment with statins (Section 2.3 Additional file 3).

##### Hypertension management

As the preoperative use of an antagonist of renin-angiotensin system (ACE inhibitor or angiotensin receptor blocker) in people undergoing CABG is associated with decreased in-hospital mortality, unless contraindicated, the OCTOPuS practitioner considered initiating an ACE inhibitor or angiotensin receptor blocker. Further agents were added in accordance with NICE guidance (Section 2.4 Additional file 3).

##### Weight management

Obesity and being overweight are associated with poor surgical outcomes. The OCTOPuS practitioner provided diet and exercise advice to all participants with a BMI >25 Kg/m^2^ (Section 2.5 and 2.6 in the Additional file 3).

##### Exercise

Specific exercise advice was offered to all participants.

##### Smoking cessation

All participants were encouraged and supported to stop smoking (Section 2.7 Additional file 3).

##### Support from spouses, other relatives or friends

The involvement of friends and family may make it easier for the participant to make the changes in medication, diet and activity more sustainable over the weeks that the intervention is delivered. Therefore, where possible, these supporters were involved in the initial and follow-up consultations (Section 2.8 Additional file 3).

### Outcome measures

The primary end-point of the study was to produce a manualised intervention, which is acceptable to patients and their healthcare professionals that could be used in a multicentre randomised controlled trial in UK cardiothoracic centres.

The following data were collected at baseline: height, weight, waist circumference, sex, age, diabetes type and duration, HbA_1c_, smoking status, blood pressure, lipid profile (total cholesterol, HDL cholesterol, triglycerides), renal function, current medication.

HbA_1c_, weight, waist circumference, smoking status, blood pressure and lipid profile were also collected on admission for surgery. Length of hospital admission was recorded. We assessed which components of the intervention were used and the number of cancelled operations. Information about adverse events was collected.

### Qualitative sub-study

Detailed, qualitative interviews with participants and healthcare professionals were undertaken to explore perceptions and experiences of the intervention and how it might be improved. Participants were interviewed 2–3 weeks after recruitment once they had received all information materials. An inductive approach was used which enabled simultaneous data collection and analyses with findings identified in early interviews iteratively informing discussions in subsequent interviews. A thematic approach was used to analyse the data, the purpose of which was to assess and understand, patterns and experiences, which cut across different people’s accounts and the reasons for these. Key aspects of the analysis included (a) comparisons between participants’ interviews to identify differences in their perceptions, experiences and behaviours, and the reasons for these; and (b) comparison of participant and health professional accounts to identify similarities and differences in their understandings and any potential impact on diabetes self-management practices, and the reasons for these. The interviews also explored participants’ information and support needs and whether, and in what ways, the intervention and follow-up care could be changed or improved.

### Statistics and data analysis

#### Sample size

Formal sample size calculation was not undertaken since the aims were primarily to assess the acceptability of the intervention to participants and healthcare professionals. It was expected that the intervention development would require approximately 20–30 participants based on previous experience of a similar trial where a more complex intervention was developed [31].

#### Data analysis

Feasibility data analysis evaluated the recruitment rate, acceptability of trial procedures and process variables. Other outcomes (participant characteristics, HbA_1c_, components of the intervention used, time from surgery to discharge and cancelled operations) were analysed as *n* (%) for categorical variables or median (IQR) for continuous variables to ensure the safety of patients and allow for refinement of the intervention.

#### Governance

University Hospital Southampton NHS Foundation Trust sponsored the study (RHM MED1368). The trial was registered with ISRCTN (ISRCTN10170306) on 10 May 2018. The day-to-day management of the trial was co-ordinated through the Southampton Clinical Trials Unit, and oversight was maintained by the Trial Steering Committee.

## Results

### Recruitment

**Fig. 1:**
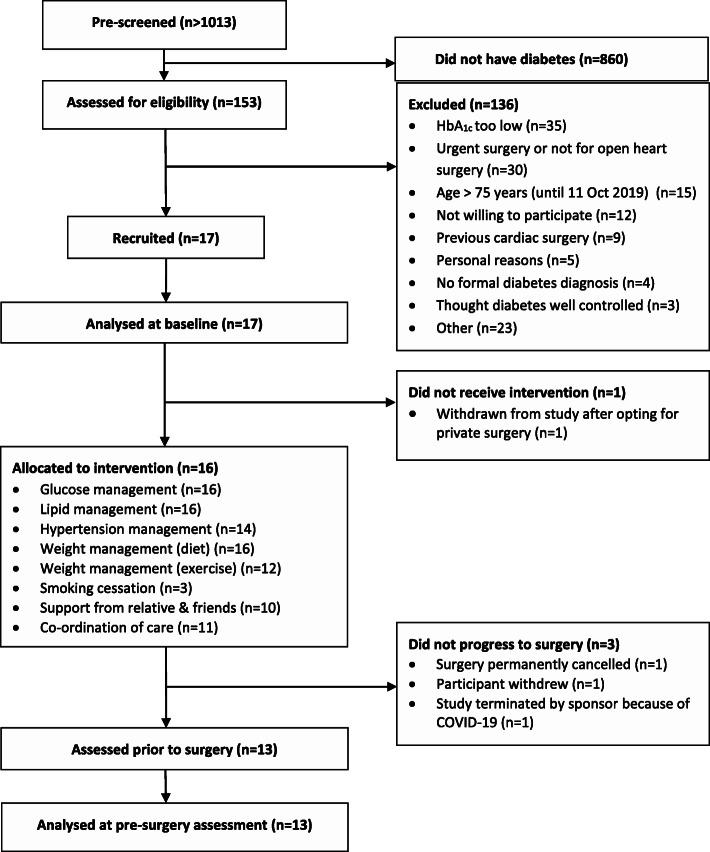
CONSORT flow diagram

The study opened to recruitment on 1 March 2019 and closed on 12 March 2020. During this time, over 1013 adults were referred to cardiothoracic outpatients clinics in Southampton (Fig. 1), of whom 153 (~15%) were reported to have diabetes. One hundred and thirty-six people did not meet the eligibility criteria or were unwilling to participate. The commonest reasons for ineligibility were HbA_1c_ below 53 mmol/mol, age >75 years (until the protocol was amended) and a clinical indication for urgent surgery. Seventeen (13% of those with diabetes) people were recruited. Recruitment was halted on 12 March 2020 because of the COVID-19 pandemic and the cessation of elective cardiac surgery. The study was not re-opened post-COVID because the OCTOPuS intervention had worked well, and only minimal changes to the manual were required, and therefore the objective of the study had been achieved.

The baseline characteristics of the 17 people recruited are shown in Table 1.

**Table 1:** Demographics and clinical assessments at baseline and pre-surgery

Characteristic	Baseline	Pre-surgery	Change from baseline to pre-surgery^**1**^
*N*	17	13	-
Patient gender			
*Male*	11 (65%)	-	-
*Female*	6 (35%)	-	-
Age	65 (62, 72)	-	-
Type of diabetes			
*Type 1*	3 (18%)	-	-
*Type 2*	14 (82%)	-	-
Diabetes duration (years)	12 (8, 18)	-	-
Smoking status^2^			
*Never smoked*	4 (24%)	-	*Stopped smoking:*
*Ex-smoker*	13 (76%)	-	0 (0%)
*Current smoker*	0 (0%)	-	
Waist circumference (cm)^3^	105.3 (103.4, 114)	98.3 (35.9, 112.2)	−4.1 (−5.3, −2.0)
Weight (kg)	84.4 (81.8, 93.6)	81.6 (63.8, 94.1)	−2.5 (−5, −1)
BMI (kg/m^2^)	31.6 (27.9, 32.6)	30.6 (28.9, 31.6)	−1.0 (−1.7, −0.4)
Systolic blood pressure (mmHg)	124 (120, 134)	124 (117, 136)	5 (−4, 14)
Diastolic blood pressure (mmHg)	78 (68, 82)	74 (65, 79)	1 (−4, 3)
Total cholesterol (mmol)^4^	4.2 (3.6, 5.1)	2.4 (0, 3.9)	−0.7 (−3.0, −0.1)
HDL cholesterol (mmol)^4^	1.2 (1.1, 1.5)	0.9 (0, 1.1)	−0.2 (−0.9, −0.1)
Triglycerides (mmol)^4,5^	1.8 (1.1, 2.7)	1.1 (0, 1.2)	−1 (−1.4, 0.1)
HbA1c (mmol/mol)^4^	68 (64, 77)	61 (56, 65)	−10 (−13, −3)
HbA1c (DCCT%)^4^	8.4% (8.0%, 9.2%)	7.7% (7.2%, 8.1%)	−1.0% (−1.2%, −0.2%)
Renal function eGFR	73 (55, 84)	60 (20, 80)	−2 (−5, 0)
No. of OCTOPuS support contacts received	-	4 (3, 7)	-
Time from baseline to pre-surgery assessment (weeks)	-	10.6 (5.6, 11.9)	-
Time from surgery to discharge (days)	-	7 (6, 9)	-

Categorical data are presented as *n* (%) and continuous data as median (IQR)

^1^Within-person change from baseline to pre-surgery assessment

^2^As reported although 3 participants were enrolled on smoking cessation intervention

^3^*n* = 5 (39%) missing at pre-surgery assessment

^4^*n* = 3 (23%) missing at pre-surgery assessment (bloods not taken)

^5^*n* = 1 (6%) missing at baseline

### Retention

One participant withdrew prior to receiving the OCTOPuS intervention, having decided to have his cardiac surgery in a private hospital outside Southampton. Sixteen participants received the OCTOPuS intervention, and 13 proceeded to surgery. Of the remaining three, the surgery was indefinitely postponed for two (one because of COVID) and one withdrew consent from the study.

### OCTOPuS intervention

After the baseline assessment, participants had an initial consultation with an OCTOPuS-trained practitioner, who was a consultant physician, or a non-medical prescriber (consultant pharmacist or diabetes specialist nurse). Follow-up care and further support were carried out by a specialist nurse, consultant pharmacist or specialist dietitian.

The median number of OCTOPuS contacts was 4 (IQR 3–7). All OCTOPuS intervention components were used, but not all parts were used for all participants. All participants were advised about glucose, lipid and the dietary aspects of weight management. Nine of the thirteen people who finished the study started a new medication (Table 1). Two participants started insulin, five started SGLT2 inhibitors and two started a GLP-1 receptor agonist (Table 2). Fourteen participants were advised about blood pressure management, twelve about exercise and three about smoking. Ten participants were advised how to seek support from friends and relatives and eleven how to obtain support from local medical services.

**Table 2:** Medication

Medication	Baseline (*n*=17)	Pre-surgery (*n*=13)	Changes in medication from baseline to pre-surgery assessment				
			Started	Stopped	Dose reduced	Dose increased	Medication type changed
Diabetes medication							
*Insulin*	7 (41%)	8 (62%)	2 (15%)	0 (0%)	4 (31%)	1 (8%)	1 (8%)
*Metformin*	13 (76%)	10 (77%)	0 (0%)	0 (0%)	1 (8%)	0 (0%)	0 (0%)
*Sulfonylurea*	3 (18%)	0 (0%)	0 (0%)	2 (15%)	0 (0%)	0 (0%)	0 (0%)
*SGLT2 Inhibitor*	3 (18%)	7 (54%)	4 (31%)	0 (0%)	0 (0%)	0 (0%)	1 (8%)
*DPP4 inhibitor*	3 (18%)	2 (15%)	0 (0%)	0 (0%)	0 (0%)	0 (0%)	0 (0%)
*GLP-1 receptor agonist*	1 (6%)	2 (15%)	2 (15%)	1 (8%)	0 (0%)	0 (0%)	0 (0%)
Other medication							
*Statin*	14 (82%)	12 (92%)	0 (0%)	0 (0%)	0 (0%)	0 (0%)	2 (15%)
*ACE inhibitor*	7 (41%)	3 (23%)	0 (0%)	2 (15%)	0 (0%)	0 (0%)	0 (0%)
*ARB*	4 (24%)	1 (8%)	0 (0%)	3 (23%)	0 (0%)	0 (0%)	0 (0%)

The median time between recruitment and surgery was 10.6 (IQR 5.6, 11.9) weeks. The median duration of the admission for surgery was 7 (IQR 6, 9) days.

There were no serious adverse events related to the OCTOPuS intervention. One participant had three cardiac arrests before surgery and was treated urgently with angioplasty instead of surgery. One participant had a prolonged postoperative hospitalisation because of his previous complex medical history.

### Qualitative interviews

Six participants completed the interview pre-surgery, four of whom also completed post-surgery interviews. These interviews ranged in duration from 15 to 30 min (mean 21 min). Participants reported that joining the trial improved their fitness and glucose levels prior to the operation. Participants communicated a good understanding of the purpose of the intervention. However, while most said they were aware of a relationship between diabetes management and surgical outcomes, they expressed limited knowledge on this topic. Some participants provided brief anecdotal commentary when asked about their understanding of the relationship (e.g. ‘I had an operation on my sinus’ and I had to keep it as low as I could for that’), while one participant reported never having thought about the relationship before.

Participants appeared motivated to make changes and improve their health. Most had previously received advice about how to use a blood glucose meter and act on the results, and several participants reported monitoring their glucose four times per day. In most cases, participants were monitoring more frequently and in a more structured manner than prior to the trial. Additionally, three participants reported starting a new medication. Furthermore, three participants mentioned that they had already lost weight or improved their glucose levels; two participants attributed these changes to new medications, while one participant attributed weight loss to dietary changes.

Among participants who had received advice from healthcare professionals in the trial, most found this advice to be helpful and felt confident that they could make the advised changes. One participant discussed a helpful call with the dietitian, who informed them to increase iron intake. A different participant mentioned that following a healthcare professional’s recommendation to reduce the insulin dose led to fewer hypoglycaemic episodes. Furthermore, participants reported that they found the intervention information sheets comprehensible and informative.

Participant interviews following surgery identified several positive factors including weight loss, greater understanding of diabetes and dietary factors and eating a healthier diet. One participant said they learned to think differently about their diabetes and things that impact blood glucose. They stated that they could ‘see what diabetes is doing to you now, it didn’t show before, it was a silent illness.’ All participants expressed willingness to continue with the behavioural changes they had made during the intervention.

The three healthcare professionals (consultant diabetologist, dietitian and consultant pharmacist) held generally favourable views of the intervention and were supportive of the holistic approach as well as the use of phone calls to provide regular support to participants. Healthcare professionals believed that the ability to receive specialist support was a major benefit for participants, as many had not previously had access to one. Accordingly, the healthcare professionals felt this was an opportunity to help patients they would not normally see. While healthcare professionals felt participants generally understood the demands of the intervention, they expressed some concern about patients’ limited knowledge regarding the relationship between diabetes management and surgical outcomes. Healthcare professionals described how patients often struggled to recognise the link between their diabetes and other conditions, suspecting GPs were not regularly conveying this information to their patients.

Despite these concerns, healthcare professionals were optimistic that participants would be motivated to make behavioural changes. They felt participants were driven by their circumstances and the high stakes involved in major surgery. Additionally, healthcare professionals noted that participants had agreed to participate in the study and therefore were more likely to be motivated than individuals who had declined. Overall, healthcare professionals generally felt that the participants could cope with the demands of the trial.

Healthcare professionals reported that participants had achieved positive outcomes, including weight loss and improved blood glucose. They were optimistic that the intervention had the potential to reduce participants’ length of stay after surgery while improving quality of life.

Responses from participants and healthcare professionals aligned across many topics covered in the interviews, such as participants’ understanding of the intervention and participants’ motivation to make changes. There was slight discrepancy between healthcare professional and participant perspectives on the challenges involved. Healthcare professionals generally felt that lifestyle changes and weight loss would be the most difficult for participants. However, when asked about whether they felt confident that they could make advised lifestyle changes, most participants said that they could do so and did not communicate any concerns.

### Process evaluation

The OCTOPuS intervention was modified in several ways based on the feedback and experience obtained throughout the pilot study. Initially we had anticipated that the first OCTOPuS visit would be made on the same day as the cardiothoracic outpatients appointment to maximise the time available to improve the diabetes management and to obviate the need for an extra hospital visit. However, this arrangement was not feasible because it meant reserving clinic space and diabetes professional time when this may not be needed. It was also difficult to predict how long the cardiothoracic consultation would last; sometimes extensive pre-surgery investigations were needed, and this could leave the patient exhausted and not in a fit state to receive information about diabetes management.

Initially, we envisaged that all people with diabetes would be seen in one cardiothoracic clinic to smooth referrals to the OCTOPuS intervention. Again, this proved impractical, not least because many referrals did not contain information about a diagnosis of diabetes.

In order to overcome these challenges, we created a dedicated weekly OCTOPuS clinic, to which participants could be referred. Many patients were willing to attend this additional appointment but some people who lived at a distance to the hospital declined.

Two further changes were made to the visit schedule. Based on participant feedback, the manual was modified to reduce the frequency of contacts once the diabetes management had been optimised and no further changes were possible to improve the clinical state from at least once a fortnight to a minimum of once every 6 weeks. This reduced participant burden and freed up the healthcare professional team.

We added a final postoperative contact in response to participant feedback to improve the continuity of diabetes care after discharge. During the qualitative study, it became apparent that the participants valued the interaction with the OCTOPuS practitioner and felt that this came to an abrupt end when the person was admitted for surgery. While the participants recognised that ongoing contact with the OCTOPuS practitioner was not possible, they expressed a desire for one postoperative contact to provide a management plan as care returned to primary care.

The COVID-19 pandemic has resulted in a shift towards remote consultations for all diabetes care. Our qualitative interviews demonstrated that the participants found remote consultations acceptable and valued the support provided. The flexibility and timing were favourably received as well as the discussion and advice received on the calls. There were some initial teething problems with sending data, but these were quickly resolved with later participants experiencing smooth data transfer and timely response. Considering these comments, we modified the manual to allow the first assessment to be undertaken remotely as well as follow-up reviews.

### Clinical assessments

Formal statistical comparisons were not made because of the design of the study. In those who completed the study, the median HbA_1c_ was 10 mmol/mol (IQR −13, -3) lower prior to surgery than at baseline. There was also a 2.5 kg reduction in body weight (IQR −5, −1 kg) and a −4.1 cm (−5.3, −2.0) reduction in waist circumference but no change in blood pressure, lipid profile or renal function. Three participants had enrolled on a smoking cessation intervention prior to the study, but no participants were smoking at baseline.

## Discussion

There is an urgent need to improve the surgical outcomes for people with diabetes, and one way of doing so is by optimising their clinical state prior to admission for surgery. This study has shown that it is possible to develop a clinical pathway to improve diabetes management prior to admission. The intervention involved regular contact between a specialist diabetes team and the person with diabetes in the time between listing for surgery and the operation. Although waiting times are usually viewed unfavourably, this intervention made a virtue of the delay.

The intervention was well received by patients and was easy to implement by the diabetes specialist team. During the study, however, a few changes were made to the prototype intervention. The biggest change was the creation of a dedicated once weekly OCTOPuS clinic; although many patients attended this additional appointment, it could disadvantage people living at a distance from the hospital. For the main trial, we will therefore incorporate the possibility of remote consultation. This may also be needed for more local patients in light of the changes in clinical practice because of the COVID-19 pandemic. Although we originally envisaged that the OCTOPuS practitioner could be any clinically qualified health care worker with expertise in diabetes, during the pilot trial, we recognised the value of the first consultation being undertaken by a prescriber. Where the initial assessment is undertaken by someone who is unable to prescribe, if changes in medication are needed, they would need to liaise with someone who could prescribe.

Given the clear evidence that elevated HbA_1c_ is associated with poor surgical outcomes, a key goal of the intervention was to lower HbA_1c_ by at least 5 mmol/mol, which is the minimal clinically important difference recognised by most guidelines. Our pilot data showed that the mean HbA_1c_ in our participants was 10 mmol/mol lower after the intervention and, if confirmed in a larger study, would be clinically significant. This reduction was similar to the 11 mmol/mol HbA_1c_ reduction seen with the Bournemouth intervention, despite the lower mean baseline HbA_1c_ of our participants. The lower baseline HbA_1c_ was unexpected but may reflect differences between people on the waiting list for cardiac surgery compared with general and orthopaedic surgery. Despite previous studies, such as the E-CABG registry showing higher HbA_1c_ [5], it is possible that the Joint British Diabetes Societies guidelines [6] have promoted greater awareness of the need for better glycaemic management in people with diabetes awaiting surgery. On the other hand, we met several patients who had elevated HbA_1c_ who declined to participate because they thought their glycaemic management was good enough.

When our study was conceived, we set an upper age limit of 75 years because we were concerned that overaggressive glucose lowering in people above this age may provoke hypoglycaemia. However, as the study progressed, we became aware that this excluded a significant number of potential participants. In hindsight, this should have been anticipated as the profile of people referred for coronary artery bypass grafting has changed. More older people with comorbidities, of which diabetes is a major contributor, are being referred [3]. We therefore removed the upper age limit for two reasons; the first was to increase recruitment, but more importantly, we recognised that if the intervention improves surgical outcomes, it is important that people over the age of 75 years are not excluded from its benefits.

The study was stopped prematurely because of the COVID-19 epidemic, and it is unclear whether further required changes may have emerged with more participants. However, the manual was always envisaged to be a ‘living document’ that could be updated as clinical guidelines and diabetes management change.

### Future research plans

Following its development and the demonstration that it is possible to deliver the intervention with clinically relevant reductions in HbA_1c_, it is important to test whether the intervention, when delivered in multiple centres, leads to not only similar reductions in HbA_1c_ but also improved surgical outcomes in people with diabetes undergoing cardiothoracic surgery. Recruitment is frequently a major issue for trials of this nature. Expanding the age range of participants will make recruitment easier as well as more equitable. Although the use of scrutinising cardiothoracic outpatient appointment lists and contacting patients ahead of their appointment worked well, there were several challenges associated with doing so. Many referral letters did not contain details of the diabetes diagnosis while other participants believed that further improvement in their diabetes management was unnecessary despite an elevated HbA_1c_. The necessity for recruitment on the day of the cardiothoracic surgical appointment was negated to a certain extent by the creation of a dedicated OCTOPuS clinic which did not run at the same time as the cardiothoracic surgical appointment. This would allow a more traditional approach to recruitment for those whose diabetes was not disclosed in the referral letter. The experience of our OCTOPuS practitioners will be used to train sites in the delivery of the intervention to maximise its benefits.

Although the main purpose of this pilot study was to prepare for our main outcome trial, the study demonstrates that preop interventions from the point of listing for surgery are possible, even when liaising across different specialties. Although this pilot study addressed diabetes in people awaiting cardiothoracic surgery, these findings could be extended to other long-term medical conditions, e.g. COPD, and other elective surgery, e.g. orthopaedic joint replacement.

## Conclusion

We have developed a feasible and acceptable manualised intervention to improve diabetes management in people with diabetes prior to cardiothoracic surgery. The clinical and cost-effectiveness of this intervention will now be tested in a multicentre randomised controlled trial in cardiothoracic centres across the UK.

## Supplementary Information

**Additional file 1.** Original trial protocol.

**Additional file 2.** Medline search strategies for Review 1 and Review 2.

**Additional file 3.** Final OCTOPuS manual.

## Data Availability

Anonymous data will be available for request from 3 months after publication of the article, to researchers who provide a completed Data Sharing request form that describes a methodologically sound proposal, for the purpose of the approved proposal and if appropriate, signed a Data Sharing Agreement. Data will be shared once all parties have signed relevant data sharing documentation, covering SCTU conditions for sharing and if required, an additional Data Sharing Agreement from Sponsor. Proposals should be directed to ctu@soton.ac.uk.
