# Supplementary material for: Developing an intervention to optimise the outcome of cardiac surgery in people with diabetes: the OCTOPuS pilot study
Source: Pilot Feasibility Stud. 2021 Aug 17;7:157. doi: 10.1186/s40814-021-00887-z (PMC8368047; doi:10.1186/s40814-021-00887-z)
Supplement: Supplementary file 1 — Additional file 1. Original trial protocol. [file 40814_2021_887_MOESM1_ESM.pdf]

Optimising Cardiac Surgery ouTcOmes in People with diabeteS – Intervention Development Study

Version 2 01-Nov-2018

SPONSOR: University Hospital Southampton NHS Foundation Trust
COORDINATING CENTRE: Southampton Clinical Trials Unit

ISRCTN reference:	ISRCTN10170306
Ethics reference number:	18/SC/0508
Sponsor reference number:	RHM MED1368
Funder reference number:	16/25/12
ICD10	[Insert]

Protocol authorised by:

**Name:**	Professor Richard Holt	**Role:**	Chief Investigator
**Signature:**		**Date:**	
**Name:**	Professor Gareth Griffiths	**Role:**	Director of SCTU
**Signature:**		**Date:**	
**Name:**	Mikayala King	**Role:**	On behalf of Sponsor
**Signature:**		**Date:**	

MAIN STUDY CONTACT

Chief Investigator and Medical Expert: Professor Richard Holt

The Institute of Developmental Sciences (IDS Building)
MP887 University of Southampton
Southampton General Hospital
Tremona Road
Southampton SO16 6YD UK

Tel: 023 8120 4665
Email: R.I.G.Holt@soton.ac.uk

STUDY COORDINATION CENTRE

For general study and clinical queries e.g. participant queries, study supplies, data collection, please contact in the first instance:

OCTOPuS Clinical Trial Manager(s): Tel: 023 8120 5154

Email: OCTOPuS@soton.ac.uk

Address: Southampton Clinical Trials Unit
Southampton General Hospital
Tremona Road
SOUTHAMPTON
SO16 6YD

Tel: 023 8120 5154
Fax: 0844 774 0621
Email: ctu@soton.ac.uk
Web: www.southampton.ac.uk/ctu

SPONSOR

University Hospital Southampton NHS Foundation Trust is the research sponsor for this study. For further information regarding sponsorship conditions, please contact the Director or Research and Development at:

Address: R&D Department
University Hospital Southampton NHS Foundation Trust
SGH, Level E, Laboratory & Pathology Block, SCBR, MP 138
Tremona Road
SOUTHAMPTON
SO16 6YD

Tel: 023 8120 4989
Fax: 023 8120 8678
Web: www.uhs.nhs.uk

CO-INVESTIGATOR(S)

Co-Investigators can be contacted via the Trial Coordination Centre.

Dr Andrew Cook	University of Southampton
Dr Mayank Patel	University Hospital Southampton NHS Foundation Trust
Dr Helen Partridge	Royal Bournemouth and Christchurch Hospitals NHS Foundation Trust
Mr Philip Newland- Jones	University Hospital Southampton NHS Foundation Trust
Mr Theodore Velissaris	University Hospital Southampton NHS Foundation Trust
Mr Kareem Salhiyyah	University Hospital Southampton NHS Foundation Trust
Professor Joanne Lord	University of Southampton
Professor Katharine Barnard	Bournemouth University
Dr Amy Whitehead	University of Southampton
Mr Stephen McGinn	PPI
Mr Sunil Ohri	University Hospital Southampton NHS Foundation Trust

FUNDER

This study is funded by NIHR Health Technology Assessment (16/25/12).

Protocol Information

This protocol describes the OCTOPuS study and provides information about procedures for entering participants. The protocol should not be used as a guide for the treatment of other non- study participants; every care was taken in its drafting, but corrections or amendments may be necessary. These will be circulated to investigators in the study, but sites entering participants for the first time are advised to contact Southampton Clinical Trials Unit to confirm they have the most recent version.

Compliance

This study will adhere to the principles of Good Clinical Practice (GCP). It will be conducted in compliance with the protocol, in accordance with current Data Protection Regulations and all other regulatory requirements, as appropriate.

SCHEDULE OF OBSERVATIONS AND PROCEDURES FOR PILOT OF INTERVENTION 10

1 INTRODUCTION 12

1.1 BACKGROUND 12

1.2 RATIONALE AND RISK BENEFITS FOR CURRENT STUDY 12

2 STUDY OBJECTIVES 13

2.1 OCTOPUS INTERVENTION DEVELOPMENT 13

3 STUDY DESIGN 15

3.1 PRIMARY ENDPOINT 15

3.2 SECONDARY ENDPOINTS 15

3.3 DEFINITION OF END OF STUDY 16

4 SELECTION AND ENROLMENT OF PARTICIPANTS 16

4.1 CONSENT 16

4.2 INCLUSION CRITERIA 16

4.3 EXCLUSION CRITERIA 16

4.4 SCREENING FAILURES 17

4.5 REGISTRATION/RANDOMISATION PROCEDURES 17

4.6 CONTRACEPTION 17

5 STUDY OBSERVATIONS AND PROCEDURES 17

5.1 SCREENING PROCEDURES 17

5.2 STUDY PROCEDURES 17

5.3 FOLLOW UP 18

5.4 DEVIATIONS AND SERIOUS BREACHES 18

5.5 STUDY DISCONTINUATION 18

5.6 WITHDRAWAL 18

5.7 PROHIBITED AND RESTRICTED THERAPIES DURING THE STUDY 19

5.8 BLINDING AND PROCEDURES FOR EMERGENCY UNBLINDING 19

6 SAFETY 19

6.1 DEFINITIONS 19

6.2 SERIOUSNESS 20

6.3 CAUSALITY 20

6.4 EXPECTEDNESS 21

6.5 REPORTING PROCEDURES 21

6.6 SCTU RESPONSIBILITIES FOR SAFETY REPORTING TO REC 22

7 STATISTICS AND DATA ANALYSES 22

7.1 SAMPLE SIZE 22

7.2 QUALITATIVE INTERVIEWS 23

8 REGULATORY 23

8.1 CLINICAL TRIAL AUTHORISATION 23

9 ETHICAL CONSIDERATIONS 23

9.1 SPECIFIC ETHICAL CONSIDERATIONS 24
9.2 ETHICAL APPROVAL 24
9.3 INFORMED CONSENT PROCESS 24
9.4 CONFIDENTIALITY 24
10 SPONSOR 25
10.1 INDEMNITY 25
10.2 FUNDING 25
10.3 AUDITS AND INSPECTIONS 25
11 STUDY OVERSIGHT GROUPS 25
11.1 TRIAL MANAGEMENT GROUP (TMG) 25
11.2 TRIAL STEERING COMMITTEE (TSC) 26
12 DATA MANAGEMENT 26
13 MONITORING 27
13.1 CENTRAL MONITORING 27
13.2 CLINICAL SITE MONITORING 27
13.3 SOURCE DATA 27
14 RECORD RETENTION AND ARCHIVING 27
15 PUBLICATION POLICY 28
16 REFERENCES 29
17 APPENDICES 31
17.1 APPENDIX 1- PROGRESS GRADING 31
18 SUMMARY OF SIGNIFICANT CHANGES TO THE PROTOCOL 33

LIST OF ABBREVIATIONS

AE	Adverse Event
CRF	Case Report Form
CTCAE	Common Terminology Criteria for Adverse Events
DMEC	Data Monitoring and Ethics Committee
GCP	Good Clinical Practice
IDSG	Intervention Development Steering Group
ISF	Investigator Site File
ITU	Intensive Treatment Unit
MHRA	Medicines and Healthcare products Regulatory Agency
NCI	National Cancer Institute
REC	Research Ethics Committee
SAE	Serious Adverse Event
SCTU	Southampton Clinical Trials Unit
TMF	Trial Master File
TMG	Trial Management Group
TSC	Trial Steering Committee

KEYWORDS
Diabetes, Intervention, Cardiac, Cardiothoracic, Surgery, Out-patient

STUDY SYNOPSIS

**Short title/Acronym:**	OCTOPuS
**Full title:**	Optimising Cardiac Surgery ouTcOmes in People with diabeteS

**Study Phase:**	III
**Population:**	Adults (18-75 yrs) with sub-optimally controlled type 1 or type 2 diabetes (HbA_{1c} > 53 mmol/mol) undergoing elective cardiac surgery, who are clinically able to wait at least 3 months for their surgery.
**Primary Objective:**	This overarching aim of the project is to investigate whether an outpatient based intervention, delivered in the weeks running up to elective major cardiac surgery, can improve outcomes for people with sub-optimally controlled diabetes.<br><br>The specific aim of the pilot study is to test whether an adaptation of an intervention, which has been used for several years for patients at Bournemouth hospital, is acceptable and feasible for a broader UK cardiothoracic population.<br><br>The primary objective of the pilot study is to produce a manualised intervention and associated training package, which is acceptable to patients and can be used in a multicentre randomised controlled trial involving at least 8 cardiothoracic centres across the UK.
**Rationale:**	There are currently two important uncertainties in the management of people with sub-optimally controlled diabetes undergoing intermediate and major surgery; how to improve diabetes management in the weeks leading up to an elective procedure, and whether that improved management is reflected in improved outcomes post-surgery. This project will develop an outpatient intervention to be delivered to people with sub-optimally controlled diabetes in need of elective cardiac surgery. If the intervention is acceptable to patients and healthcare professionals, we will then conduct a randomised controlled trial to assess whether the intervention can bring down their HbA_{1c}, and improve clinical outcomes. The randomised controlled trial will be subject to a separate ethics submission
**Study Design:**	A single centre, feasibility study to develop an outpatient manualised intervention and associated training package, which is acceptable to patients and healthcare professionals.
**Sample size :**	20-30
**Treatment/Intervention:**	An outpatient based intervention delivered over approximately 12 weeks prior to surgery

**URL for Database:**	[Insert]

**Primary Study Endpoints:**	The development of an acceptable and deliverable outpatient intervention

**Secondary Study Endpoints:**	Pilot data on:<br>• Basic demographics of who the intervention is delivered to<br>• Actual time from surgery to actual discharge from hospital<br>• Change in weight between randomisation and surgery<br>• HbA_{1c} immediately preoperative.<br>• Change in HbA_{1c} between baseline and immediately preoperative<br>• Change in blood glucose between baseline and immediately preoperative<br>• Operations cancelled for sub-optimal glycaemic control<br>• Which components of the intervention are used
**Total Number of Sites:**	1

STUDY SCHEMA

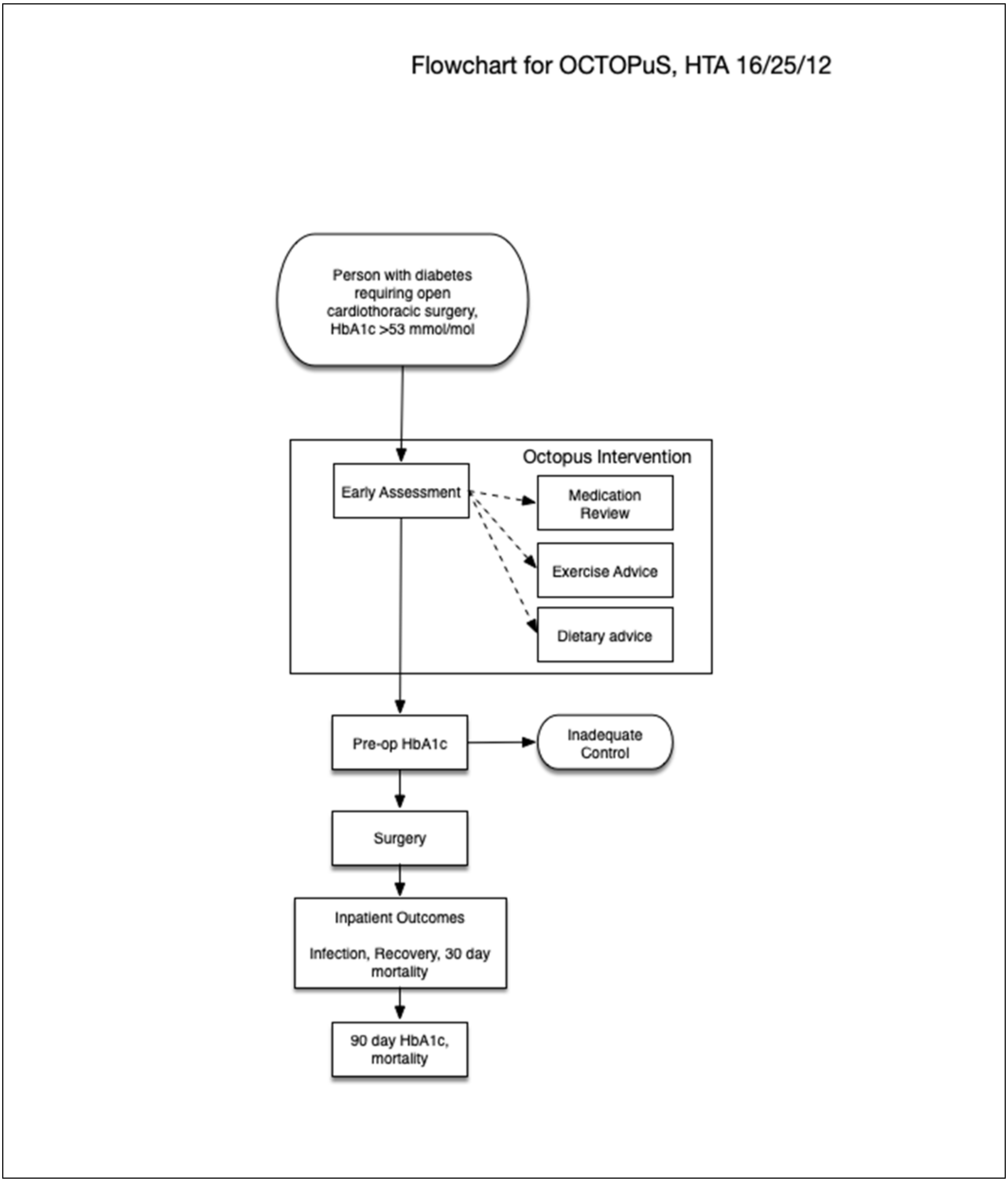

SCHEDULE OF OBSERVATIONS AND PROCEDURES FOR PILOT OF INTERVENTION

Visit:	Pre-Screening	Screening & Baseline	Support call Week 2	Support call Week 4	Support call Week 6	Support call Week 8	Support call Week 10	Support call Week 12[a]	Surgery	Discharge
Time (days):										
Notes review	X									
Informed Consent		X								
Eligibility evaluation (incl. pregnancy test)	X	X								
Medical History (incl. smoking status, diabetes and current medications)		X							X	
Physical Exam (incl height, weight and waist circumference)		X							X	
Vital Signs (incl. BP)		X							X	
Biochemistry (incl HbA_{1c}, blood glucose and renal function)		X							X	
Intervention		X								
Intervention support phone call[a] (incl. review of diary card and components of intervention utilised)			X	X	X	X	X	X		
Surgery									X	
Adverse Events		X	X	X	X	X	X	X	X	X

Qualitative Interview		**X**								**X**[b]
Time to discharge										**X**
Surgery details										**X**

[a] further support calls may be needed at fortnightly intervals if the surgery is delayed beyond 12 weeks from listing. These phone consultations will not produce data to be captured, but will prompt phone discussion regarding adherence to the intervention and/or during interviews.
[b] interviews will be conducted within 6 weeks of surgery.

NB: The Participant/legal representative is free to withdraw consent at any time without providing a reason. When withdrawn, the participant will continue to receive standard clinical care. Follow up data will continue to be collected unless the participant/legal representative has specifically stated that they do not want this to happen.

1 INTRODUCTION

1.1 BACKGROUND

There are approximately 4 million people living with diagnosed and undiagnosed diabetes mellitus in the UK (1). Since 1996, the number of people diagnosed with diabetes has increased from 1.4 million to approximately 3.5 million. Diabetes increases the risk of cardiovascular disease by approximately 2-fold after adjustment for other cardiovascular risk factors. Ischaemic heart disease is by far the leading cause of death in people with diabetes accounting for approximately two thirds of all deaths in those aged >65 years. Coronary heart disease tends to be more diffuse and progresses more rapidly in people with diabetes which may explain why up to 35% of those presenting for elective cardiac revascularisation have diabetes (2). Sub-optimal glycaemic control increases the risk of wound and chest infections, renal impairment and death, especially following cardiac surgery (3-7).

This project will address whether a pre-operative out-patient non-doctor delivered intervention to improve diabetes management improves cardiac surgical outcomes for people with diabetes.

The increasing number of people with diabetes will increase the demand for cardiac surgery in the future. These patients have longer lengths of hospital stay and higher re-admission rates, placing a large financial burden on the NHS. If the pre-operative intervention is successful in improving glycaemic control, this may reduce the complication rate and improve the clinical outcomes. It may also prove cost effective and even cost saving.

The Joint British Diabetes Societies for in-patient care provided recommendations to improve the management of adults with diabetes undergoing surgery. As sub-optimal peri-operative glycaemic control is associated with an increased risk of all surgical complications, the guidelines recommend improving glycaemic control to optimise surgical outcomes.

Over the past 5 years the diabetes team at the Royal Bournemouth Hospital have worked to optimise the surgical experience of people with diabetes. Using a nurse-led outpatient intervention, delivered around 3 months before surgery to people with sub-optimally controlled diabetes, they achieved a reduction in HbA_{1c} from 85 mmol/mol at first referral to 74 mmol/mol on admission for surgery. This has been associated with a reduced length of stay from a mean of 5.9 days to a mean of 3 days, while the length of stay for those without diabetes remained constant at 5 days. Other work has shown the practicality of improving HbA_{1c} over a period of weeks in primary care (8).

We intend to adapt and manualise this intervention so it can be used in other UK surgical centres. We will then test its acceptability and feasibility for people with diabetes awaiting cardiac surgery and the healthcare professionals delivering the intervention compared with usual care.

1.2 RATIONALE AND RISK BENEFITS FOR CURRENT STUDY

There are currently two important uncertainties in the management of people with sub-optimally controlled diabetes undergoing intermediate and major surgery; 1) how to improve diabetes management in the weeks leading up to an elective procedure, and 2) whether that improved management is reflected in improved outcomes post-surgery (9).

Practice is therefore varied, with current UK guidelines recommending a delay to surgery to allow for improved diabetes control if HbA_{1c} is above 69 mmol/mol; whereas in the USA guidance recommends considering delaying surgery at an HbA_{1c} above 53 mmol/mol. The current NICE guidelines recognise this as an evidence gap (10), as do the Joint British Diabetes Societies (3).

The project described here will address both questions, in the specific setting of cardiothoracic surgery. We will develop an outpatient intervention to be delivered to patients with sub-optimally controlled diabetes in need of elective cardiac surgery. If the intervention is deemed acceptable to patients and health professionals, we will test the effectiveness of the intervention in a large scale RCT which will be subject to a separate ethics application.

2 STUDY OBJECTIVES

This study aims to investigate whether an outpatient based intervention, delivered in the weeks running up to elective major cardiac surgery, can be developed that is acceptable to patients and health professionals.

To do this an intervention, which has been used for several years for patients at Bournemouth Hospital, was adapted to make it suitable for a broader UK cardiothoracic population. We will use the approach recommended by the MRC's complex intervention evaluation framework (11).

- In partnership with people with diabetes, patients who have undergone cardiac surgery, and clinicians, we will adapt the Bournemouth intervention to be applicable to a more diverse cardiothoracic population.
- We will test the pilot intervention in groups of 4-6 people with diabetes who are undergoing cardiac surgery and refined the intervention in an iterative process over 3-4 cycles
- Through qualitative and psychosocial research, we will assess whether the intervention when scaled up for the main RCT, is appropriate for and acceptable to clinicians and people with sub-optimally controlled diabetes.

This project has 2 key elements:

1. Intervention development
    - A nine month development period where the OCTOPuS intervention will be refined and manualised, and an initial training package developed.

2. Qualitative Interviews
    - Qualitative and psychosocial research to investigate the patient experience of the intervention, and assess its acceptability to patients and clinicians.

2.1 OCTOPUS INTERVENTION DEVELOPMENT

The first draft of the OCTOPuS intervention has been developed by refining the service currently used in Bournemouth. This has involved compiling the constituent components in the context of cardiac patients. This has been overseen by an intervention development steering group (IDSG), comprising trial investigators, staff from Bournemouth who have

delivered the intervention there, and an anaesthetist. Further input has been obtained from the trial oversight committees, the in-patient diabetes team in Southampton and people with diabetes and experience of cardiac surgery drawn from the study PPI advisory group and local branch of Diabetes UK.

2.1.1 Rapid Literature Review

This has built on current recommendations from the Joint British Diabetes Societies (3), and ensures that all potential elements of the intervention are considered. The literature review comprised two parts; the first review was designed to identify factors that predict poor surgical outcomes in people with diabetes. The purpose of this review was to ensure that the OCTOPuS intervention did not ignore other important risk factors beyond glucose management. The second review was to identify studies that reported pre-surgical admission interventions.

2.1.2 Qualitative Data Collection

Clinicians from cardiothoracic and diabetes disciplines from Bournemouth and Southampton have examined the Bournemouth intervention and findings from the systematic review to discuss the key components of the prototype intervention.

A survey has been developed and distributed to determine current practice for management of people with diabetes in cardiac surgery centres. This will help inform how the OCTOPuS intervention and trial will fit into current clinical practice.

2.1.3 Intervention Manualisation

The learning from the two previous work packages has been synthesised into a first draft of an intervention manual. This will be given to a purposive sample of people with diabetes (sampled to capture diversity in terms of age, occupation, socio-economic status). In addition, discussion regarding the intervention with members of the multidisciplinary cardiothoracic surgery team as well as primary care and specialist diabetes healthcare professionals will take place in order to gain a broad range of key stakeholder views.

This manual will be reviewed and adapted in an iterative process after discussion with our PPI steering group and with clinicians from Southampton, Bournemouth and members of the Trial Steering Committee.

The first draft of the manual includes the review, assessment and management of the following:

- Glucose management (lifestyle and diabetes medication)
- Lipid management
- Hypertension management
- Weight management
    - Diet
    - Exercise
- Smoking cessation
- Support from relative and friends
- Co-ordination of care

2.1.4 Training Package Development

In parallel to developing the OCTOPuS manual, a training package will be developed for OCTOPuS to allow its dissemination across all trial sites. This training package will be an output of the study should the OCTOPuS intervention be shown to be effective.

The same IDSG will oversee the development of the training package, receiving feedback from the OCTOPuS practitioners who are delivering the intervention.

2.1.5 Progression rules for moving to main trial

If a manualised intervention and associated training package cannot be provided, which is acceptable to patients, it will be impossible to deliver the main study. The TSC will review a report of stage 1 from the Trial Management Group, and make a recommendation to stop or proceed.

3 STUDY DESIGN

A single centre, feasibility study to develop an outpatient manualised intervention and associated training package, which is acceptable to people with sub-optimally controlled type 1 or type 2 diabetes undergoing elective cardiothoracic surgery and health professionals.

3.1 PRIMARY ENDPOINT

To produce a manualised intervention and associated training package, which is acceptable to patients and their healthcare professionals that can be used in a multicentre randomised controlled trial involving at least 8 cardiothoracic centres across the UK.

3.2 SECONDARY ENDPOINTS

The following data will be collected on paper CRF pre and post intervention to allow us to report descriptive data about those who have received the intervention:

- Height
- Weight
- Waist circumference
- Sex
- Age
- Diabetes type and duration
- HbA1c
- Smoking status
- Blood pressure
- Blood glucose
- Current medication
- Components of intervention used
- Time from surgery to actual discharge from hospital
- Operations cancelled for sub-optimal glycaemic control

Participants will also be asked to keep a food diary and monitor their blood glucose 4 times per day in the 3 days prior to each phone call to aid discussion about future diabetes management and during interview. These data will not be recorded for trial purposes.

3.3 DEFINITION OF END OF STUDY

The end of trial is defined as the date after the last patient has their last interview where all data required to answer the research question are captured.

4 SELECTION AND ENROLMENT OF PARTICIPANTS

4.1 CONSENT

Consent to enter the study must be sought from each participant only after a full explanation has been given, an information leaflet offered and time allowed for consideration. Signed participant consent should be obtained. The right of the participant to refuse to participate without giving reasons must be respected. After the participant has entered the study the clinician remains free to give alternative treatment to that specified in the protocol at any stage if he/she feels it is in the participant's best interest, but the reasons for doing so should be recorded. In these cases the participants remain within the study for the purposes of follow-up and data analysis. All participants are free to withdraw at any time from the protocol treatment without giving reasons and without prejudicing further treatment.

A traditional approach to seeking consent to take part in the research at the outpatient appointment, and giving patients at least 24 hours to consider the trial and discuss with their social support before making a decision and then returning for their first OCTOPuS consultation presents a challenge. First, it adds a visit to a potentially distant hospital, and second it does not reflect how this intervention would be used should it be rolled out into practice as we would expect the OCTOPuS consultation to usually take place on the same day as the cardiothoracic outpatient appointment. This is necessary to maximise any opportunity to improve diabetes management prior to surgery.

Therefore the study will adopt an approach of informing patients about the study similar to that used by Foss et al (12), who demonstrated that patients receiving telephone based counselling about a trial showed similar levels of comprehension to those being counselled face-to-face. See section 5.1 below for screening and consent procedures.

Upon completion of the informed consent form, a copy will be given to the patient, a copy stored in the patient's medical notes, a copy sent to the SCTU and the original filed in the Investigator Site File. The SCTU copy should be emailed to uhs.sctu@nhs.net using a secure nhs.net email address to allow for central monitoring.

4.2 INCLUSION CRITERIA

- Adults (aged 18-75 yrs) with sub-optimally controlled type 1 or type 2 diabetes. Sub-optimal control is defined as an HbA_{1c} > 53 mmol/mol using a near patient test at the cardiothoracic outpatients appointment where the decision to proceed to surgery is made.
- Awaiting elective cardiac surgery, where it is anticipated the delay before surgery will be at least 3 months.
- Ability to give informed consent.
- Ability to interact with the study documentation and processes.

4.3 EXCLUSION CRITERIA

- Malignancy
- Pregnancy
- Previous cardiac surgery

- Known Haemoglobinopathies
- Other illnesses or conditions that would preclude engagement with the OCTOPuS intervention

4.4 SCREENING FAILURES

Screen failures will be recorded on the 'patient screening log'. This is completed for all patients who have been considered for the study and is faxed or emailed monthly to the OCTOPuS Trial Team on 0844 774 0621 or octopus@soton.ac.uk

4.5 REGISTRATION/RANDOMISATION PROCEDURES

After consent, participants will be given a sequential trial ID from a provided list. A registration form will be sent to Southampton CTU to centrally document study registrations.

4.6 CONTRACEPTION

There are no study-specific requirements for the use of contraception.

5 STUDY OBSERVATIONS AND PROCEDURES

5.1 SCREENING PROCEDURES

Outpatient appointment lists will be scrutinised by research nurses a month ahead of appointments, to identify patients who may meet the study eligibility criteria. For patients who appear eligible for the OCTOPuS study, an information sheet explaining the trial will be sent. Around two weeks before the outpatient appointment a member of the site trial team will telephone the prospective participant to discuss the study, thus allowing sufficient time for reflection and discussion before the outpatient appointment. The information sheet will also include contact details to opt out if the patient does not want to be contacted about the trial. This will allow patients who are eligible for the study at the outpatient appointment to provide consent, and receive their first OCTOPuS consultation, on the same day.

At the outpatient appointment where a decision to proceed to surgery is made, the treating surgeon will remind eligible patients about the trial. If the patient wishes to take part, a more detailed interview with a research nurse will follow, where the study can be discussed in depth according to the needs of the patient, and final exclusion criteria checked (e.g. pregnancy status) and written consent given.

5.2 STUDY PROCEDURES

Patients will have an initial consultation with an OCTOPuS trained health professional (OCTOPuS Practitioner), who may be a nurse, pharmacist, or other appropriately trained person. In this consultation the patient's diabetes management will be discussed, as well as the likely benefits that improved glycaemic control will provide in the run up to surgery. The practitioner and patient will agree a number of actions, tailored to the individual needs and ability. These are likely to include:

- A graded exercise regimen. This may be completely self-delivered, or alternatively by joining a local appropriate exercise scheme – such as a 'health walk'. There is a general consensus among the cardiothoracic community that limited exercise can be

allowed prior to surgery. This needs to be individualised for each patient, and should not provoke symptoms of angina or breathlessness. The usual format of exercise suggested is walking on the flat, for short, frequent, episodes.

- Dietary advice, possibly supplemented by a consultation with a dietitian
- Medication review, which may lead to the introduction of insulin for people with type 2 diabetes.
- Specific advice about managing expectations, understanding facilitators to achieve change and overcoming barriers to improve medical and psychosocial outcomes

Patients will receive regular review with the OCTOPuS practitioner, probably by telephone, at least once a fortnight. This will be an opportunity to offer encouragement and support, and address any issues which have arisen for the patient.

Where necessary the OCTOPuS practitioner will liaise with local services, e.g. the patient's GP or a dietitian, to facilitate delivery.

5.3 FOLLOW UP

Patients will be interviewed within 6 weeks of receiving their surgery.
Follow up will be as per local practice.

5.4 DEVIATIONS AND SERIOUS BREACHES

Any study protocol deviations/violations and breaches of Good Clinical Practice occurring at sites should be reported to the SCTU and the local R&D Office immediately. SCTU will then advise of and/or undertake any corrective and preventative actions as required.

All serious protocol deviations/violations and serious breaches of Good Clinical Practice and /or the study protocol will immediately be reported to the regulatory authorities and other organisations, as required in the Medicines for Human Use (Clinical Trials) Regulations 2004, as amended.

5.5 STUDY DISCONTINUATION

In consenting to the study, participants have consented to the study intervention and data collection. Participants may be discontinued from the study procedures at any time.

5.5.1 Reasons for study discontinuation

Participants may be discontinued from the study in the event of:

- Clinical decision, as judged by the Principal Investigator or Chief Investigator
- In the event the trial is discontinued due to the interim analysis (as outlined in Section 7.3)

Full details of the reason for study discontinuation should be recorded in the eCRF and medical record.

5.6 WITHDRAWAL

The participant / legal representative is free to withdraw consent from the study at any time without providing a reason.
Investigators should explain to patients the value of remaining in study follow-up and allowing this data to be used for trial purposes. Where possible, patients who have

withdrawn from study treatment should remain in follow-up as per the trial schedule. If patients additionally withdraw consent for this, they should revert to standard clinical care as deemed by the responsible clinician. It would remain useful for the study team to continue to collect standard follow-up data and unless the patient explicitly states otherwise, follow-up data will continue to be collected.

Details of study discontinuation (date, reason if known) should be recorded in the CRF and medical record.

5.7 PROHIBITED AND RESTRICTED THERAPIES DURING THE STUDY

There are no prohibited or restricted therapies during this study.

5.8 BLINDING AND PROCEDURES FOR EMERGENCY UNBLINDING

Due to the nature of the interventions in this trial there will be no blinding of participants or investigators.

6 SAFETY

6.1 DEFINITIONS

Adverse Event (AE): any untoward medical occurrence in a participant or clinical study participant which does not necessarily have a causal relationship with study treatment or participation.
An AE can therefore be any unfavourable and unintended sign (including an abnormal laboratory finding), symptom, or disease temporally associated with the study treatment or participation (regardless of causality assessments).

Serious Adverse Event (SAE) is any untoward medical occurrence or effect that:

- **Results in death**
- **Is life-threatening** – *refers to an event in which the participant was at risk of death at the time of the event; it does not refer to an event which hypothetically might have caused death if it were more severe*
- **Requires hospitalisation, or prolongation of existing hospitalisation**
- **Results in persistent or significant disability or incapacity**
- **Is a congenital anomaly or birth defect**
- Other important medical events***.

*'life-threatening' in the definition of 'serious' refers to an event in which the patient was at risk of death at the time of the event; it does not refer to an event which hypothetically might have caused death if it were more severe.

**Hospitalisation is defined as an inpatient admission, regardless of length of stay, even if the hospitalisation is a precautionary measure for continued observation. Hospitalisations for a pre-existing condition, including elective procedures that have not worsened, do not constitute an SAE.

***Other important medical events may also be considered serious if they jeopardise the participant or require an intervention to prevent one of the above consequences.
Note: It is the responsibility of the PI or delegate to grade an event as 'not serious' (AE) or 'serious' (SAE).

6.2 SERIOUSNESS

A complete assessment of the seriousness must always be assessed by a medically qualified doctor who is registered on the delegation of responsibility log; this is usually the investigator.

All adverse events that fulfil the criteria definition of 'serious' in protocol section 6.1, must be reported to SCTU using the Serious Adverse Event Report Form – Non-CTIMP. Specific exceptions to this (as listed below) should be recorded as AEs rather than SAEs.

All SAEs must be reported immediately by the PI at the participating centre to the SCTU.

6.2.1 Exceptions:

For the purposes of this study, the following SAEs **do not** require reporting to SCTU using the Serious Adverse Event Report Form – Non-CTIMP:

- Hospitalisations for elective treatment of a pre-existing condition

6.3 CAUSALITY

A complete assessment of the causality must always be assessed by a medically qualified doctor who is registered on the delegation of responsibility log; this is usually the investigator.

If any doubt about the causality exists the local investigator should inform the SCTU who will notify the Chief Investigator. Other clinicians may be asked for advice in these cases.

Relationship	Description	Event Status
**Unrelated**	There is no evidence of any causal relationship	Not related to treatment
**Unlikely**	There is little evidence to suggest there is a causal relationship (e.g. the event did not occur within a reasonable time after administration of the study treatment). There is another reasonable explanation for the event (e.g. the participant's clinical condition, other concomitant treatment).	Not related to treatment
**Possibly**	There is some evidence to suggest a causal relationship (e.g. because the event occurs within a reasonable time after administration of the study treatment). However, the influence of other factors may have contributed to the event (e.g. the participant's clinical condition, other concomitant treatments).	Related and expected SAE/ Related and unexpected SAE
**Probably**	There is evidence to suggest a causal relationship and the influence of other factors is unlikely.	Related and expected SAE/ Related and unexpected SAE
**Definitely**	There is clear evidence to suggest a causal relationship and other possible contributing factors can be ruled out.	Related and expected SAE/ Related and unexpected SAE

In terms of event status; **Not related to treatment** would highlight that the SAE is not related to the trial treatment. **Related and expected** SAE would signify that the SAE is

related to the trial treatment and is expected (according to the list of expected events listed in the protocol). **Related and unexpected SAE** would be classified as an SAE which is related to the trial treatment and is unexpected in terms of the events listed in the protocol.

In the case of discrepant views on causality between the Investigator and others, SCTU will classify the event as per the worst case classification I and where applicable the Ethics Committee will be informed of both opinions within the required timelines.

6.4 EXPECTEDNESS

Expectedness assessments are made against the list of expected events below:

6.4.1 Expected Adverse Events:

- Minor musculoskeletal aches and pains
- Myocardial infarction
- Known adverse effects of medications used in the intervention as per the SPCs.

The nature or severity of should be considered when making the assessment of expectedness. If these factors are not consistent with the current information available then the AE should be recorded as ’unexpected’.

6.5 REPORTING PROCEDURES

All adverse events should be reported.

Depending on the nature of the event, the appropriate reporting procedures below should be followed. A flowchart will be provided to aid in the reporting procedures.

6.5.1 Reporting Details

A SAE for Non-CTIMPs Form should be completed for all SAEs and faxed to SCTU within 24 hours of site becoming aware of the event.

Complete the SAE form and fax or email a scanned copy of the form with as many details as possible to the SCTU together with anonymised relevant treatment forms and investigation reports.
Or
Contact the SCTU by phone for advice and then fax or email a scanned copy of the completed SAE form.

SAE REPORTING CONTACT DETAILS

Please email or fax a copy of the SAE form to SCTU within 24 hours of becoming aware of the event

Fax: 0844 774 0621 or Email: ctu@soton.ac.uk

FAO: Quality and Regulatory Team

For further assistance: Tel: 023 8120 4138 (Mon to Fri 09:00 – 17:00)

Additional information should be provided as soon as possible if the event has not resolved at the time of reporting.

6.5.2 Follow Up and Post- study SAEs

The reporting requirement for all AEs and SAEs affecting participants applies for all events occurring up to 30 days following cardiac surgery.

All unresolved adverse events should be followed by the investigator until resolved, the participant is lost to follow-up, or the adverse event is otherwise explained. At the last scheduled visit, the investigator should instruct each participant to report any subsequent event(s) that the participant, or the participant's general practitioner, believes might reasonably be related to participation in this study. The investigator should notify the study sponsor of any death or adverse event occurring at any time after a participant has discontinued or terminated study participation that may reasonably be related to this study.

6.5.3 Non-serious AEs

All adverse events should be recorded in the relevant CRF and submitted to SCTU.

6.5.4 Pre-existing Conditions

Medically significant pre-existing conditions (those which are present prior to informed consent) should not be reported as an AE unless the conditions worsens during the trial. The condition, however, must be reported on the Baseline CRF. Any adverse events which occur after informed consent taken should be recorded on the AE CRF as per safety reporting section.

6.5.5 Serious Adverse Events

All SAEs should be reported within 24 hours of the local site becoming aware of the event. The SAE Non-CTIMP Form asks for nature of event, date of onset, severity, corrective therapies given, outcome, causality (i.e. unrelated, unlikely, possible, probably, definitely) and expectedness. The responsible investigator should assign the causality and expectedness of the event with reference to the events listed in Section 6.4.1 . The event term should be in accordance with the latest version of MedDRA and grades given in accordance with the NCI CTCAE v5, Additional information should be provided as soon as possible if the event has not resolved at the time of reporting.

6.6 SCTU RESPONSIBILITIES FOR SAFETY REPORTING TO REC

SCTU will notify the necessary competent authorities of all **Related and Unexpected** SAEs occurring during the study within 15 days.

SCTU submit all safety information to the REC in annual progress report.

7 STATISTICS AND DATA ANALYSES

7.1 SAMPLE SIZE

It is expected that the intervention development will require approximately 20 participants. This number is based on cycles of 4-6 patients with up to 4 iterative cycles. This figure is based on previous experience of the Chief Investigator from a similar trial where a more complex intervention was developed (14).

Feasibility data analysis will be descriptive: recruitment rates, participant characteristics, acceptability of trial procedures and process variables.

7.2 QUALITATIVE INTERVIEWS

Qualitative interviews with participants and healthcare professionals will explore perceptions and experiences of the intervention and how it might be improved. Treatment fidelity will be maintained by including all content to be covered on checklists, assessed by intervention access/usage and examining any diversions from the protocol.

Data analysis will commence as soon as data collection begins. Meetings will be held between the team to discuss preliminary findings, make refinements to the topic guides if required and to agree on a coding frame. A thematic approach will be used to analyse the data, the purpose of which is to look for, and understand, patterns and experiences which cut across different people's accounts and the reasons for these. Key aspects of the analysis will include: (a) comparisons between participants' interviews to identify differences in their perceptions, experiences and behaviours, and the reasons for these; (b) comparison of participant and health professional accounts to identify similarities and differences in their understandings and any potential impact on diabetes self-management practices; (c) cross-comparison of participants' accounts to identify common issues and experiences as well differences between subgroups of participants (e.g. men versus women, participants of different ages etc.), and the reasons for these.

The interviews will also explore participants' information and support needs and whether, and in what ways, the intervention and follow-up care could be changed or improved.

7.2.1 Quality procedures

Several quality procedures will be used to increase the validity and credibility. Procedures to be used are for instance: using a member check, the use of several data collection methods (triangulation), using a reflexive diary, doing the analyses by two researchers and the use of 'thick descriptions'.

8 REGULATORY

8.1 CLINICAL TRIAL AUTHORISATION

This study is not considered to be a clinical trial of a medicinal product, so clinical trial authorisation from the UK Competent Authority the Medicines and Healthcare products Regulatory Agency (MHRA) is not applicable.

9 ETHICAL CONSIDERATIONS

The study will be conducted in accordance with the recommendations for physicians involved in research on human participants adopted by the 18th World Medical Assembly, Helsinki 1964 as revised and recognised by governing laws and EU Directives. Each participant's consent to participate in the study should be obtained after a full explanation has been given of treatment options, including the conventional and generally accepted methods of treatment. The right of the participant to refuse to participate in the study without giving reasons must be respected.

After the participant has entered the study, the clinician may give alternative treatment to that specified in the protocol, at any stage, if they feel it to be in the best interest of the

participant. However, reasons for doing so should be recorded and the participant will remain within the study for the purpose of follow-up and data analysis according to the treatment option to which they have been allocated. Similarly, the participant remains free to withdraw at any time from protocol treatment and study follow-up without giving reasons and without prejudicing their further treatment.

9.1 SPECIFIC ETHICAL CONSIDERATIONS

Our proposal to randomise patients on the same day as the decision to proceed to surgery is made may prove controversial. However, it is believed that this is entirely justified as (i) this is a pragmatic approach, which reflects how the intervention would be used in routine practice, (ii) it is more convenient for patients, relieving them on an additional journey to a potentially remote hospital, and (iii) this approach to consent has been used previously within the European Union (12). All patients will have an opportunity to discuss the study face-to-face with a clinician before randomisation, and will of course able to withdraw should they change their mind.

Participants will be reassured that all personally identifiable data collected during the course of the research will be kept strictly confidential, and non-identifiable data will be shared in accordance with the University of Southampton policies. All patient data will be anonymised and stored on a database in accordance with current Data Protection Regulations. We will also seek the patient's permission to inform their general practitioner that they are taking part in this study. Documentation relating to clinical trials managed by Southampton CTU is retained for 15 years after notification of the trial's end.

9.2 ETHICAL APPROVAL

The study protocol has received the favourable opinion of a Research Ethics Committee or Institutional Review Board (IRB) in the approved national participating countries.

9.3 INFORMED CONSENT PROCESS

Informed consent is a process that is initiated prior to an individual agreeing to participate in a study and continues throughout the individual's participation. In obtaining and documenting informed consent, the investigator should comply with applicable regulatory requirements and should adhere to the principles of GCP.

Discussion of objectives, risks and inconveniences of the study and the conditions under which it is to be conducted are to be provided to the participant by appropriately delegated staff with knowledge in obtaining informed consent with reference to the patient information leaflet. This information will emphasise that participation in the trial is voluntary and that the participant may withdraw from the trial at any time and for any reason. The participant will be given the opportunity to ask any questions that may arise and provided the opportunity to discuss the study with family members, friend or an independent healthcare professional outside of the research team and time to consider the information prior to agreeing to participate.

9.4 CONFIDENTIALITY

SCTU will preserve the confidentiality of participants taking part in the study. The investigator must ensure that participant's anonymity will be maintained and that their identities are protected from unauthorised parties. On CRFs participants will not be identified by their names, but by an identification code.

10 SPONSOR

SCTU, Chief Investigator and other appropriate organisations have been delegated specific duties by the Sponsor and this is documented in the trial task allocation matrix.

The duties assigned to the study sites (NHS Trusts or others taking part in this study) are detailed in the Non-Commercial Agreement.

10.1 INDEMNITY

For NHS sponsored research HSG (96) 48 reference no.2 applies. If there is negligent harm during the clinical study when the NHS body owes a duty of care to the person harmed, NHS Indemnity covers NHS staff, medical academic staff with honorary contracts, and those conducting the study. NHS Indemnity does not offer no-fault compensation and is unable to agree in advance to pay compensation for non-negligent harm. Ex-gratia payments may be considered in the case of a claim.

10.2 FUNDING

This study is funded by the National Institute for Health Research Health Technology Assessment Programme.

10.2.1 Site payments

The payments assigned to the study sites (NHS Trusts or others taking part in this study) are detailed in the Non-Commercial Agreement.

This study is automatically eligible for the NIHR portfolio. This enables Trusts to apply to their comprehensive local research network for service support costs, if required.

10.2.2 Participant payments

Participants will not be paid for participation in this study.

10.3 AUDITS AND INSPECTIONS

The study may be participant to inspection and audit by UHS (under their remit as Sponsor), SCTU (as the Sponsor’s delegate) and other regulatory bodies to ensure adherence to the principles of GCP, Research Governance Framework for Health and Social Care, applicable contracts/agreements and national regulations.

11 STUDY OVERSIGHT GROUPS

The day-to-day management of the trial will be co-ordinated through the SCTU and oversight will be maintained by the Trial Management Group, the Trial Steering Committee and the Data Monitoring and Ethics Committee.

11.1 TRIAL MANAGEMENT GROUP (TMG)

The TMG is responsible for overseeing progress of the study, including both the clinical and practical aspects. The Chair of the TMG will be the Chief Investigator of the study.

The OCTOPuS TMG charter defines the membership, terms of reference, roles, responsibilities, authority, decision-making and relationships of the TMG, including the timing of meetings, frequency and format of meetings and relationships with other trial committees.

11.2 TRIAL STEERING COMMITTEE (TSC)

The TSC act as the oversight body on behalf of the Sponsor and Funder. The TSC will meet in person at least yearly and have at least one further teleconference meeting during the year. The majority of members of the TSC, including the Chair, should be independent of the study.

The OCTOPuS TSC charter defines the membership, terms of reference, roles, responsibilities, authority, decision-making and relationships of the TSC, including the timing of meetings, frequency and format of meetings and relationships with other trial committees.

12 DATA MANAGEMENT

Participant data will be entered onto paper CRF and sent to Southampton CTU retained in accordance with current Data Protection Regulations. The PI is responsible for ensuring the accuracy, completeness, and timeliness of the data entered and that each CRF is signed.

The participant data is pseudo anonymised by assigning each participant a participant identifier code which is used to identify the participant during the study and for any participant- specific clarification between SCTU and site. The site retains a participant identification code list which is only available to site staff.

The Informed Consent Form will specify the participant data to be collected and how it will be managed or might be shared; including handling of all Patient Identifiable Data (PID) and sensitive PID adhering to relevant data protection law.

Only the Investigator and personnel authorised by them should enter or change data in the CRFs. When requested, laboratory data must be transcribed, with all investigator observations entered into the CRF. The original laboratory reports must be retained by the Investigator for future reference.

A Data Management Plan (DMP) providing full details of the study specific data management strategy for the trial will be available and a Trial Schedule with planned and actual milestones, CRF tracking and central monitoring for active trial management created.

Data queries will be manually raised by the study team, if required.

At the end of the study after all queries have been resolved, the PI will confirm the data integrity by signing the appropriate CRF. The CRFs will be archived according to SCTU policy.
Data may be requested from the Data Access Committee at SCTU. Request will be considered on a monthly basis.

13 MONITORING

13.1 CENTRAL MONITORING

Data stored at SCTU will be checked for missing or unusual values (range checks) and checked for consistency within participants over time. Any suspect data will be returned to the site in the form of data queries. Data query forms will be produced at SCTU from the trial database and sent either electronically or through the post to a named individual (as listed on the site delegation log). Sites will respond to the queries providing an explanation/resolution to the discrepancies and return the data query forms to SCTU. The forms will then be filed along with the appropriate CRFs and the appropriate corrections made on the database. There are a number of monitoring features in place at SCTU to ensure reliability and validity of the trial data, which are detailed in the trial monitoring plan.

13.2 CLINICAL SITE MONITORING

Monitoring will be completed as per the trial monitoring plan. However, it is not expected that there will be any onsite monitoring as part of the intervention development.

13.2.1 Source Data Verification

On receipt of a written request from SCTU, the PI will allow the SCTU direct access to relevant source documentation for verification (taking into account data protection regulations). Access should also be given to study staff and departments (e.g. pharmacy).

The participants' medical records and other relevant data may also be reviewed by appropriate qualified personnel independent from the SCTU appointed to audit the study, including representatives of the Competent Authority. Details will remain confidential and participants' names will not be recorded outside the study site.

13.3 SOURCE DATA

Source documents are where data are first recorded, and from which participants' CRF data are obtained. These include, but are not limited to, hospital records (from which medical history and previous and concurrent medication may be summarised), clinical and office charts, laboratory and pharmacy records, diaries, microfiches, radiographs, and correspondence.

14 RECORD RETENTION AND ARCHIVING

Trial documents will be retained in a secure location during and after the trial has finished.

The PI or delegate must maintain adequate and accurate records to enable the conduct of the study to be fully documented and the study data to be subsequently verified. After study closure the PI will maintain all source documents and study related documents. All source documents will be retained for a period of 15 years following the end of the study.

Sites are responsible for arching the ISF and participant's medical records.

The Sponsor is responsible for archiving the TMF and other relevant documentation.

15 PUBLICATION POLICY

Data from all centres will be analysed together and published as soon as possible.

Individual investigators may not publish data concerning their patients that are directly relevant to questions posed by the trial until the Trial Management Group (TMG) has published its report. The TMG will form the basis of the Writing Committee and advise on the nature of publications. All publications shall include a list of investigators, and if there are named authors, these should include the Chief Investigator, Co-Investigators, Trial Manager, and Statistician(s) involved in the trial. Named authors will be agreed by the CI and Director of SCTU. If there are no named authors then a ‘writing committee’ will be identified.

16 REFERENCES

1. Diabetes UK. Facts and Stats [Internet]. diabetes.org.uk. 2015 [cited 2016 Aug 4]. Available from: https://www.diabetes.org.uk/About_us/What-we-say/Statistics/

2. Engoren M, Habib RH, Zacharias A, Schwann TA, Riordan CJ, Durham SJ, et al. The prevalence of elevated hemoglobin A1c in patients undergoing coronary artery bypass surgery. Journal of Cardiothoracic Surgery 2008 3:1. BioMed Central; 2008 Nov 24;3(1):63.

3. Joint British Diabetes Societies for Inpatient Care. Management of adults with diabetes undergoing surgery and elective procedures: Improving standards. 2015 Sep 9;:1–84.

4. Thourani VH, Weintraub WS, Stein B, Gebhart S, Craver JM, Jones EL, et al. Influence of diabetes mellitus on early and late outcome after coronary artery bypass grafting. Ann Thorac Surg. 1999 Apr;67(4):1045–52.

5. Risum Ø, Abdelnoor M, Svennevig JL, Levorstad K, Gullestad L, Bjørnerheim R, et al. Diabetes Mellitus and Morbidity and Mortality Risks after Coronary Artery Bypass Surgery. Scandinavian Journal of Thoracic and Cardiovascular Surgery. 2009 Jul 12;30(2):71–5.

6. Salomon NW, Page US, Okies JE, Stephens J, Krause AH, Bigelow JC. Diabetes mellitus and coronary artery bypass. Short-term risk and long-term prognosis. J Thorac Cardiovasc Surg. 1983 Feb;85(2):264–71.

7. Dhatariya K, Levy N, Kilvert A, Watson B, Cousins D, Flanagan D, et al. NHS Diabetes guideline for the perioperative management of the adult patient with diabetes. Diabet Med. 2012 Apr 1;29(4):420–33.

8. Hirst A, Dutton S, Wu O, Briggs A, Edwards C, Waldenmaier L, et al. A multi-centre retrospective cohort study comparing the efficacy, safety and cost-effectiveness of hysterectomy and uterine artery embolisation for the treatment of symptomatic uterine fibroids. The HOPEFUL study. Health technology assessment (Winchester, England). 2008 Mar 1;12(5):1–248, iii.

9. Rollins KE, Varadhan KK, Dhatariya K, Lobo DN. Systematic review of the impact of HbA_{1c} on outcomes following surgery in patients with diabetes mellitus. Clin Nutr. 2016 Apr;35(2):308–16.

10. National Guideline Centre (UK). Preoperative Tests (Update): Routine Preoperative Tests for Elective Surgery. London: National Institute for Health and Care Excellence (UK); 2016 Apr.

11. Craig P, Dieppe P, Macintyre S, Michie S, Nazareth I, Petticrew M. Developing and evaluating complex interventions: new guidance. Glasgow: Medical Research Council; 2008. 39 p.

12. Foss KT, Kjærgaard J, Stensballe LG, Greisen G. Recruiting to Clinical Trials on the Telephone - a randomized controlled trial. Trials. BioMed Central; 2016 Nov 21;17(1):552.

13. Hirst JA, Stevens RJ, Farmer AJ. Changes in HbA_{1c} level over a 12-week follow-up in patients with type 2 diabetes following a medication change. Barengo NC, editor. PLoS ONE. Public Library of Science; 2014;9(3):e92458.

14. Gossage-Worrall R, Holt RIG, Barnard K, Carey ME, Davis MJ, Dickens C, Doherty Y, Edwardson C, French P, Gaughran F, Greenwood K, Kalidindi S, Hind D, Khunti K, McCrone P, Mitvhell J, Pendlebury J, Rathod S, Shiers D, Siddiqi N, Swaby L, Wright S. STEPWISE – Structured lifestyle Education for People With SchizophrEniaL a study protocol for a randomised controlled trial. Trials (2016) 17:475.

17 APPENDICES

17.1 APPENDIX 1- PROGRESS GRADING

Table 1 – Actions to be taken depending on progress grade

Grade	Action
Green	Continue trial, keeping an eye on accrual.
Amber	Working with governance committees (TSC, TMG, PPI Committees), seek root cause for under performance. Consider whether these can be mitigated through work with organisations or individuals within the study.
Red	Review the study with governance committees, taking steps as detailed under amber, but also explicitly considering recommending study closure.

Table 2 - Progress grading time points and criteria

Assessment Point	Green	Amber	Red
After 100 patients have had surgery (50 intervention and 50 control)	HbA_{1c} reduction in intervention group > 5mmol/mol	HbA_{1c} reduction in intervention group < 5mmol/mol	HbA_{1c} reduction in intervention group not consistent with physiological effect
6 complete months after 1st recruiting site has opened	>= 8 centres have recruited at least one patient and<br>>= 50 patients have been recruited and<br>At least 10 patients have completed their OCTOPuS intervention and have either received surgery, or have had their surgery cancelled or postponed for either clinical or operational reasons.	One or two criteria in green column met	No criteria in green column met
12 complete months after 1st recruiting site has opened	>= 12 centres have recruited at least one patient and<br>and<br>At least 50 patients have completed their OCTOPuS intervention and have either received surgery, or have had their surgery cancelled or postponed for either clinical or operational reasons.	Only one criterion from green column met	No criteria on green column met
End of month 15 of recruitment	The mean recruitment rate across the trial in months 13, 14, and 15 following the opening of the first recruiting centre is compatible with completing recruitment by the end of month 27 of recruitment	The mean recruitment rate across the trial in months 13, 14, and 15 following the opening of the first recruiting centre is compatible with achieving 75% or more of target recruitment by the end of month 27	The mean recruitment rate across the trial in months 13, 14, and 15 following the opening of the first recruiting centre is not compatible with achieving at least 75% of target recruitment by the end of month 27

18 SUMMARY OF SIGNIFICANT CHANGES TO THE PROTOCOL

Protocol date and version	Summary of significant changes
V2<br>31-Oct-2018	Remove references to the main randomised controlled trial so that current protocol is for the development of the intervention alone.
