# Supplementary material for: Developing an intervention to optimise the outcome of cardiac surgery in people with diabetes: the OCTOPuS pilot study
Source: Pilot Feasibility Stud. 2021 Aug 17;7:157. doi: 10.1186/s40814-021-00887-z (PMC8368047; doi:10.1186/s40814-021-00887-z)
Supplement: Supplementary file 2 — Additional file 2. Medline search strategies for Review 1 and Review 2. [file 40814_2021_887_MOESM2_ESM.docx]

REVIEW 1

| **Ovid MEDLINE(R) Epub Ahead of Print, In-Process & Other Non-Indexed Citations, Ovid MEDLINE(R) Daily and Ovid MEDLINE(R) <1946 to Present** |
| --- |
| 1 exp Diabetes Mellitus/su [Surgery] (15223)  2 exp Postoperative Complications/ (538550)  3 ((postoperativ* or "post operativ*" or "perioperativ*" or "peri operativ*" or operativ*) adj5 complication*).tw,kw. (104648)  4 2 or 3 (597132)  5 1 and 4 (1996)  6 (outcome* and surg* and diabet*).tw. (9852)  7 (complication* and surg* and diabet*).tw. (9785)  8 (risk* and surg* and diabet*).tw. (12595)  9 or/6-8 (21686)  10 1 and 9 (2165)  11 5 or 10 (3553)  12 exp Diabetes Mellitus/ (418747)  13 (diabetes or diabetic*).tw,kw. (590907)  14 exp Diabetes Complications/ (131225)  15 or/12-14 (654268)  16 exp Specialties, Surgical/ (199687)  17 exp Surgical Procedures, Operative/ (3097365)  18 Elective Surgical Procedures/ (12908)  19 (surgery or surgical).tw,kw. (1674621)  20 or/16-19 (4003907)  21 15 and 20 (86359)  22 11 or 21 (86432)  23 (surg* and complicat*).ti. (17624)  24 Comorbidity/ (103505)  25 23 and 24 (236)  26 (predict* and surg* complication*).tw. (1221)  27 25 or 26 (1434)  28 Hemoglobin A, Glycosylated/ (35008)  29 ("A1c" or "HBA1c").tw,nm. (40661)  30 (hyperglyc?emia or hyperglyc?emic).tw. (57911)  31 exp hyperglycemia/ or exp hypoglycemia/ (61384)  32 (hypoglyc?emia or hypoglyc?emic).tw. (53825)  33 Blood Glucose/ (165308)  34 (glyc?emic adj control).tw. (29968)  35 or/28-34 (285059)  36 20 and 35 (26752)  37 (hypertension or hypertensive).tw. (417696)  38 hypertension/ (239080)  39 Metabolic Syndrome X/ (31773)  40 "metabolic syndrome".tw. (47314)  41 Insulin Resistance/ (55666)  42 "insulin resistance".tw. (75115)  43 exp Hyperlipidemias/ (67823)  44 hyperlipid?emi*.tw. (29509)  45 exp Obesity/ (203201)  46 (obese or obesity).tw. (270921)  47 Non-alcoholic Fatty Liver Disease/ (8603)  48 "Non-alcoholic hepatic steatosis".tw. (40)  49 (arthritis or arthritic).tw. (175484)  50 exp Arthritis/ (260051)  51 or/37-50 (1187502)  52 22 and 51 (22335)  53 exp Obesity/su (18989)  54 Hyperlipidemias/su [Surgery] (123)  55 exp Hypertension/su [Surgery] (4383)  56 Metabolic Syndrome X/su [Surgery] (206)  57 Non-alcoholic Fatty Liver Disease/su [Surgery] (103)  58 exp arthritis/su (26598)  59 or/53-58 (50063)  60 15 and 59 (3940)  61 (comorbidit* and surg* and diabet* and outcome*).tw. (1318)  62 exp Exercise/ (179865)  63 ("physical* activ*" or inactiv* or sedentary).tw. (409555)  64 "physical* fit*".tw. (8958)  65 physical exertion/ or physical fitness/ or cardiorespiratory fitness/ (87062)  66 exercise test/ or walk test/ (66201)  67 or/62-66 (649642)  68 22 and 67 (1447)  69 22 or 27 or 36 or 52 or 60 or 61 or 68 (101148)  70 limit 69 to yr="2000 -Current" (68212)  71 limit 70 to english language (61701)  72 limit 71 to (meta analysis or systematic reviews) (2460)  73 limit 71 to "reviews (maximizes specificity)" (1453)  74 72 or 73 (2607)  75 meta-analysis/ (98610)  76 meta-analysis as topic/ (18023)  77 meta-analysis.pt. (98610)  78 meta-analysis.ab. (98638)  79 metanaly*.ab. (211)  80 systematic review*.tw. (126148)  81 systematic overview*.tw. (962)  82 academic review.tw. (26)  83 cochrane database of systematic reviews.jn. (14837)  84 hand search*.tw. (6251)  85 (medline or medlars or embase or pubmed or cochrane or scisearch or psychinfo or psycinfo o psychlit or cinahl or "web of science").ab. (172741)  86 (electronic database* or bibliographic database* or online database*).ab. (26177)  87 ("methodological review" or "methodological overview").ab. (227)  88 "manual search*".ab. (3949)  89 data extraction.ab. (17127)  90 or/75-89 (310491)  91 71 and 90 (1874)  92 74 or 91 (2891)  93 Pregnancy in Diabetics/ (11292)  94 Diabetes, Gestational/ (9407)  95 "gestational diabetes".tw. (12403)  96 (diabet* adj5 pregnan*).tw. (11707)  97 or/93-96 (26432)  98 92 not 97 (2781)  99 (child* or schoolchild* or adolesc* or infant* or toddler*).ti. (1021850)  100 98 not 99 (2745) |

REVIEW 2

| **Ovid Medline Literature Search Strategy** |
| --- |
| 1 exp Diabetes Mellitus/ (375358)  2 (diabetes or diabetic*).tw,kw. (534254)  3 or/1-2 (592557)  4 (surgery or surgical).tw,kw. (1527326)  5 Elective Surgical Procedures/ (11409)  6 exp Specialties, Surgical/ (185600)  7 exp Surgical Procedures, Operative/ (2823175)  8 or/4-7 (3665666)  9 3 and 8 (77954)  10 Preoperative Care/ or Preoperative Period/ (61849)  11 Perioperative Care/ (11401)  12 ("preoperativ*" or "pre operativ*" or "perioperativ*" or "peri operativ*").tw. (331039)  13 (patient* adj2 (admission* or admit*)).tw. (71665)  14 ((pre or before or beforehand or prior or previous or advance) adj2 hospital*).tw. (13964)  15 Patient Admission/ and (pre or before or beforehand or prior or previous or advance).tw. (3994)  16 ((pre or before or beforehand or prior or previous or advance) adj2 admit*).tw. (762)  17 (preadmit* or pre admit* or preadmission or "pre admission").tw. (2186)  18 (pre hab* or prehab*).tw. (276)  19 (presurg* or pre-surg*).tw. (11471)  20 (pre adj2 (postsurg* or postoperativ*)).tw. (15108)  21 or/10-20 (464060)  22 9 and 21 (10337)  23 (glyc?emi* adj5 (control* or optimal or optimiz* or optimis* or manage* or improv* or monitor)).tw. (29627)  24 (glucose adj5 (control or optimal or optimiz* or optimis* or manage* or improv* or monitor*)).tw. (39929)  25 *Blood Glucose/ or Blood Glucose Self-Monitoring/ (48547)  26 Glycated Hemoglobin A/ (29465)  27 HbA1c.tw. (25299)  28 "h?emoglobin A1c".tw. (9019)  29 hyperglyc?emia.tw. (44789)  30 Hyperglycemia/ (24827)  31 or/23-30 (164627)  32 8 and 21 and 31 (2348)  33 22 or 32 (11288)  34 ("healthy life" or "healthy lives" or "healthy lifestyle" or "healthy life style" or "healthy living").tw. (7298)  35 exp Life Style/ (79127)  36 (exercis* or walk* or run* or jog* or cycling or aerobic* or danc* or pilates or gym or swim* or hydrotherapy or sport*).tw. (656319)  37 (preconditioning or pre conditioning or physical fitness or physically fit or physiotherapy or physical therap*).tw. (55008)  38 ((muscle or endurance or resistance or weight or strength) adj2 training).tw. (17618)  39 ((function or "functional capacity") adj2 (enhanc* or improv* or maximis*)).tw. (51163)  40 exp Exercise/ or exp Exercise Therapy/ (187595)  41 (physical adj2 (exertion or training or movement or activity or activities or endurance or condition*)).tw. (103163)  42 physical endurance/ or physical exertion/ or exp physical fitness/ (91281)  43 exp sports/ (158951)  44 Physical Therapy Modalities/ (33187)  45 physical therap*.tw. (18540)  46 exp Diet Therapy/ (48665)  47 exp Nutrition Therapy/ (91528)  48 (diet or nutrition).tw. (386721)  49 weight.tw. (719268)  50 or/34-49 (1952786)  51 33 and 50 (1896)  52 (health adj2 (educat* or information or awareness or pilot* or program* or promot* or improv* or intervention* or initiative* or empower* or strateg* or prevent* or project* or campaign* or skill*)).tw. (196648)  53 ((education$ or psychoeducational or psychotherapeutic or psychological or psychosocial or behavio?ral or cognitive) adj3 (intervention$ or program$)).ti,ab. (87363)  54 health education/ or health promotion/ or healthy people programs/ or weight reduction programs/ or patient education as topic/ or needs assessment/ (215627)  55 ((counselling or counseling) adj2 (session* or therap* or intervention* or program*)).tw. (5710)  56 exp Cognitive Therapy/ (22672)  57 CBT.tw. (8195)  58 (cognitive adj2 (therap* or intervention*)).tw. (18961)  59 ((selfcare or self-care or self-help or coping) adj2 (mechanism* or strateg* or behavio?r*)).tw. (17706)  60 exp counseling/ (39586)  61 ((nutrition or metabolic) adj2 support).tw. (3503)  62 or/52-61 (506693)  63 33 and 62 (131)  64 (intervention* or program* or educat* or pilot* or initiative* or strateg* or counsel* or protocol*).tw. (2878030)  65 33 and 64 (2666)  66 Nurse Practitioners/ (16569)  67 nutritionists/ or occupational therapists/ or pharmacists/ or physical therapists/ (15837)  68 (pharmacist* or dietician* or nutritionist or physiotherap* or "physical therap*" or "non doctor*").tw. (66794)  69 or/66-68 (88031)  70 33 and 69 (49)  71 51 or 63 or 65 or 70 (4156)  72 (exp child/ or exp infant/) not adult/ (1572611)  73 ((child* or infant* or newborn* or neonat* or toddler* or schoolchild*) not adult*).ti. (954619)  74 72 or 73 (1815106)  75 71 not 74 (4068)  76 limit 75 to english language (3669)  77 (comment or editorial or letter).pt. (1602551)  78 76 not 77 (3652) |
